# Potential Ancestral Conoidean Toxins in the Venom Cocktail of the Carnivorous Snail *Raphitoma purpurea* (Montagu, 1803) (Neogastropoda: Raphitomidae)

**DOI:** 10.3390/toxins16080348

**Published:** 2024-08-09

**Authors:** Giacomo Chiappa, Giulia Fassio, Maria Vittoria Modica, Marco Oliverio

**Affiliations:** 1 Department of Biology and Biotechnologies “Charles Darwin”, Sapienza University of Rome, Viale dell’Università 32, 00185 Rome, Italy; giulia.fassio@uniroma1.it (G.F.); marco.oliverio@uniroma1.it (M.O.); 2 Department of Biology and Evolution of Marine Organisms, Stazione Zoologica Anton Dohrn, Via Gregorio Allegri 1, 00198 Rome, Italy; mariavittoria.modica@szn.it

**Keywords:** Raphitomidae, transcriptome, conotoxin, venom duct, salivary glands, venom evolution, trophic ecology

## Abstract

Venomous marine gastropods of the superfamily Conoidea possess a rich arsenal of toxins, including neuroactive toxins. Venom adaptations might have played a fundamental role in the radiation of conoideans; nevertheless, there is still no knowledge about the venom of the most diversified family of the group: Raphitomidae Bellardi, 1875. In this study, transcriptomes were produced from the carcase, salivary glands, and proximal and distal venom ducts of the northeastern Atlantic species *Raphitoma purpurea* (Montagu, 1803). Using a gut barcoding approach, we were also able to report, for the first time, molecular evidence of a vermivorous diet for the genus. Transcriptomic analyses revealed over a hundred putative venom components (PVC), including 69 neurotoxins. Twenty novel toxin families, including some with high levels of expansion, were discovered. No significant difference was observed between the distal and proximal venom duct secretions. Peptides related to cone snail toxins (Cerm06, Pgam02, and turritoxin) and other venom-related proteins (disulfide isomerase and elevenin) were retrieved from the salivary glands. These salivary venom components may constitute ancestral adaptations for venom production in conoideans. Although often neglected, salivary gland secretions are of extreme importance for understanding the evolutionary history of conoidean venom.

## 1. Introduction

Venoms are secretions with the capacity to interfere with the physiological processes of a target organism [1,2]. In the animal kingdom, a wide range of different venoms has evolved for both defensive and offensive functions in predation and intraspecific competition [3]. Toxins, the bioactive molecules that take part in the envenomation process, are of great interest for basic and applied research due to their relevance in shaping the ecology and evolution of venomous organisms and their possible pharmacological application [4]. Venom composition is related to different trophic ecological traits, such as dietary breadth [5,6,7], predation strategy [8], diet shift [9], and intraspecific niche partitioning [4,10].

Marine gastropods include many venomous taxa, the most studied being the cone snails of the family Conidae Fleming, 1822, which employ powerful neurotoxins to incapacitate their prey or elude their predators [4,11]. The conid venom cocktail is characterised by a mixture of short neuroactive peptides named conotoxins, which are highly diversified and able to affect a wide range of vertebrate and invertebrate taxa [12]. The signal sequence and cysteine pattern of conotoxins are sufficiently conserved to achieve a sound classification system [13,14], which comprises at least 16 superfamilies [15]. In envenomation, conotoxins can enable prey capture [16,17] and play a defensive role [17] or several metabolic functions [18,19]. Neuropeptides have high therapeutic potential, making the toxin-rich venom cocktails of Conidae an interesting target for translational research [19].

Although the venom of ~120 cone snail species has been characterised (https://www.conoserver.org, accessed on 7 May 2024), the number of described conotoxins is meagre compared to the estimated total [20,21]. One of the mechanisms underlying the extraordinary diversification of conotoxins is the neofunctionalisation of genes following duplication and positive selection [5,22]. This process involves not only toxins but also toxin maturation and processing enzymes. For instance, a whole different family of chaperone protein disulfide isomerases, specialised in toxin folding, was retrieved in the venom duct of Conidae [23].

Our knowledge of neogastropod venoms is mostly limited to cone snails, and many of the adaptations underlying their toxicity are known for this family only. The secretions of a few other conoidean taxa have also been assessed, such as Terebridae and Turridae—as well as some species of superfamilies Tonnoidea, Buccinoidea, and Muricoidea—but the taxonomic coverage of predatory gastropods outside of Conidae is still very low [24,25,26]. To gain a better understanding of the evolutionary history of the venom (e.g., what ancestral innovations promoted the use of toxins? When did the expansion of toxin genes occur?), a broader taxonomic scope is needed. Given that the evolution of a venom system might have constituted a key evolutionary innovation leading to adaptive radiation in Conoidea [26,27,28], one of the best targets for such studies is certainly the most species-rich group [29,30] of Conoidea, Raphitomidae Bellardi, 1875. The family Raphitomidae includes almost 900 extant described species (WoRMS, accessed on 13 May 2024) and a probably larger portion of undescribed species [26]. According to the most recent phylogenetic reconstruction of Conoidea, whose origin is estimated to be ~138 Ma, this family emerged at ~50 Ma and is closely related to cone snails [31,32]. The type genus *Raphitoma* Bellardi, 1847 is a surprisingly complex taxon, with 57 extant named species (WoRMS, accessed on 13 May 2024), of which over a third were described in the last decade in the Mediterranean Sea [33,34,35]. Despite the potential interest in this radiation in various biological aspects (e.g., the evolution of larval development [36,37,38]), the life history and evolutionary biology of *Raphitoma* are still largely unknown.

The trophic ecology of raphitomids has not yet been determined. They are presumed to be carnivorous predators like the rest of the conoideans, also considering their foregut anatomy, which includes a toxoglossan proboscis and a venom duct, as in Conidae [30,39]. Direct observations of polychaetes as the target prey [40], indeed, matched our unpublished data from metabarcoding of the stomach content in a specimen of *Raphitoma bicolor* Risso, 1826 (NCBI accession number: PP913530), identified with the polychaete genus *Polycirrus*. The anatomical variability of raphitomids, with several taxa completely lacking a venom duct [30,39], might suggest an evolutionary pattern driven by trophic adaptations, making them an interesting model for the study of toxicity. The characterisation of Raphitomidae venom would be crucial to clarify many ecological aspects of these poorly understood species and provide an explanation for the remarkable radiation which occurred in the family. Furthermore, the characterisation of toxins produced by *Raphitoma* could reveal if those discovered in cone snails originated during the radiation of Conidae or rather constitute earlier adaptations shared with their sister lineage.

In recent years, venom research has been revolutionised by large-scale sequencing approaches, which implement next-generation platforms to gather a comprehensive dataset of transcripts in a specific tissue involved in the envenomation, like salivary or venom glands and their ducts [27,41]. When dealing with the first transcriptome of a major taxon (family, order, etc.), an approach based mainly on sequence homology could fail to retrieve most novel substances; however, modern bioinformatic pipelines retrieve toxin components adopting multiple strategies such as the detection of transmembrane domains and cysteine patterns, folding prediction, cellular localisation, and expression levels [42]. High-throughput sequencing data are subject to sequencing and bioinformatic analyses biases and tissue contamination [42,43], and the functional characterisation of transcripts (e.g., toxic vs. physiological) needs to be experimentally validated through bioactivity assays [44,45]. Furthermore, the overall venom composition may vary depending on the moment the sample is collected, e.g., after a predation event (when the animal may be replenishing the venom) vs. during a predation event (when active production may be scarce). However, despite these limitations, transcriptomic approaches have provided a huge boost to the identification of new toxins compared to proteomic assays [28]; the number of peptides putatively identified as toxins in reference databases is increasing exponentially, demanding caution in the interpretation of transcriptomic results.

In this study, we performed an in-depth characterisation of the secretome of salivary glands and venom duct of *Raphitoma purpurea*. We identified and described the main toxic components on the basis of tissue-specific expression, the scoring system of DeTox, a comprehensive pipeline for toxin discovery, and a manually curated annotation. Published data from a large set of neogastropod species, especially conids, were used as a reference to identify potential functional similarities of *Raphitoma* toxins. Our large-scale approach aimed to identify both peptides with toxic properties (with a focus on conotoxin-like peptides) and proteins responsible for toxin maturation and regulation. Finally, evidence of predatory behaviour in *Raphitoma* is provided as two additional prey taxa are identified from the gut contents using the mitochondrial marker *16S rDNA*.

## 2. Results

### 2.1. Bioinformatics Analyses

Following extraction, the RNA quantity of the carcases was 19.44–40.32 µg, with 4.9–6.2 RIN values. The RNA quantity of salivary glands and venom ducts was 19.4–269 ng, with 6.4–8 RIN. Sequencing yielded 786 million paired-end reads, 40–72 million per sample. The final assembly comprised 937,142 transcripts, with an N50 of 881.

The highest BUSCO score was retrieved for the Metazoa reference, with a 99.48% completeness score (94.76% complete and 4.72% fragmented matches), while the Mollusca score was 71.26% (66.59% complete and 4.66% fragmented matches). Bacterial-targeted analyses had the lowest completion scores. Most matching transcripts were detected in the salivary glands, followed by the venom ducts. A total of 10,671 transcript matches were found overall in the transcriptomes, of which 486 were retrieved by DeTox as candidate venom components.

**Figure 1:**
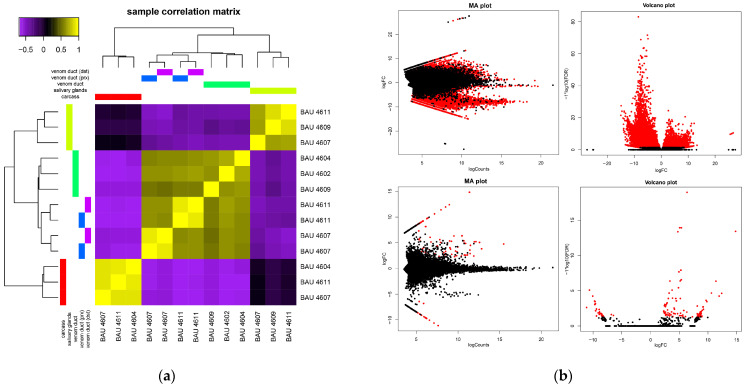
Results of the differential expression analysis; (**a**) Correlation matrix among samples, yellow colour represents high correlation; (**b**) Tissue-specific gene expression plots for venom duct vs. salivary glands (above) and distal vs. proximal venom duct (below), red dots represent differentially expressed transcripts.

DeSeq2 (Figure 1) identified 9990 differentially expressed transcripts, of which 2614 were overexpressed in the salivary glands and 7208 in the venom duct samples. Furthermore, 106 and 112 transcripts were overexpressed in the distal and proximal portions of the venom duct, respectively, compared to the whole duct, and 34 transcripts were differentially expressed between the two sections. Those overexpressed in the proximal fragment were related to cellular transport—such as actin, myosin light, and heavy chains, troponin I-like, filamin-A—and muscle contraction—such as titin-like, twitchin-like, and LIM—and did not include any putative venom component (PVC). Transcripts overexpressed in the distal fragment had very low expression levels and could not be characterised. The correlation matrix among the tissue samples did not differentiate between the two sections of the venom duct (Figure 1).

**Figure 2:**
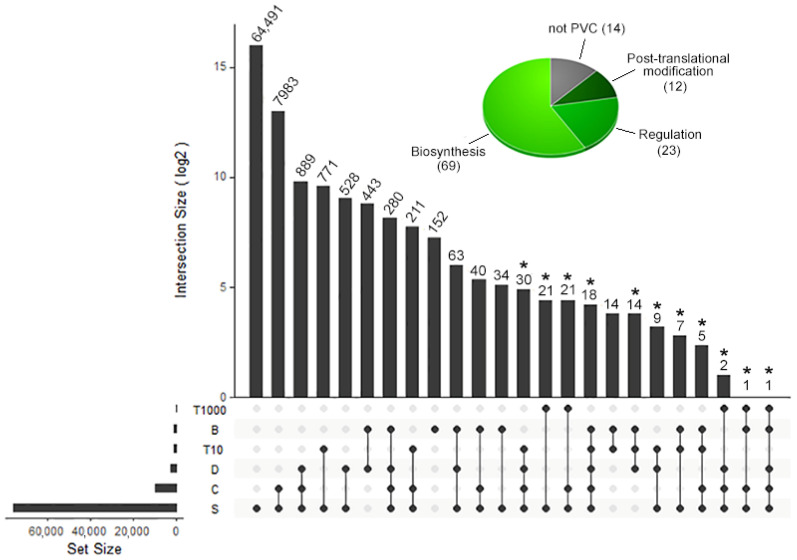
UpsetR diagram of candidate toxic components identified by Detox with relative frequencies of all score combinations. Constructed in RStudio [46]. Bars scaled with the “log2” parameter. Values above the bars represent the frequencies. The pie chart shows the results of the PVC functional annotation (marked with “*” over the upsetR diagram bars). Score legend: S = signal sequence and no transmembrane domain; C = cysteine framework; D = domain match with Pfam; B = similarity match with the reference database; T-x: TPM in salivary glands or venom duct equal to or greater than x.

A total of 74,520 transcripts were selected by the DeTox pipeline (Figure 2; Supplementary Material Table S1) as candidate venom components, and 118 passed the filtering steps based on scoring and expression level and were subsequently processed for functional annotation. After annotation, 14 transcripts were discarded as unrelated to toxic activity, resulting in 104 PVC (Table 1; Supplementary Material Table S2)—with function related to post-translational modification (*n =* 12) or regulation (*n =* 23) and biosynthesis (*n =* 69). The expression level in either the salivary glands or the venom duct was high for 47% of the PVC, medium for 11%, and low for the remaining. Several transcripts (*n* = 53) were recognised as differentially expressed between salivary glands and venom ducts, the vast majority predominantly expressed in the latter (*n* = 51). Venom duct neuropeptides represented the highest number of PVC (*n* = 49) with the highest expression levels (40 highly expressed, including ten with TPM > 10000). This group also included all the lowest DeTox scoring transcripts (“S”, *n* = 17), which were retained because of their outstanding TPM values but could not be adequately annotated.

ConoDictor identified 9530 conotoxin-like peptides in the DeTox output. After filtering, 145 transcripts (of which only 15 were among the PVC) were retained, hereafter named raphitoxins; they were ascribed to 20 putative raphitoxin families (PRF) (Supplementary Material Table S3). The classes identified were conotoxins (*n* = 105), conodipines (*n* = 6), conoCAP (*n* = 1), conkunitzins (*n* = 9), contryphan (*n* = 1), and conatokins (*n* = 6), while 17 transcripts remained unclassified. ConoPrec superfamily attribution based on the signal sequence was unsuccessful for all sequences, and no close known conotoxin was retrieved for any transcript. Following ConoDictor predictions—which were mostly not unanimous within each family—the PRF was associated with superfamilies A (*n* = 3), M (*n* = 1), O1 (*n* = 6), conkunitzin (*n* = 2), and unknown (*n* = 8). Eleven clusters had higher expression in the venom duct; two were mainly retrieved from the salivary glands, and the remaining were similarly expressed. Four PRF showed signs of gene expansion, with 10–23 transcripts each, including transcripts that were preferentially expressed in different tissues. In the two PRFs, the mature peptide length was longer than expected for neuropeptides (>300 aa). The signal sequence of 13 additional PVC not retrieved by this pipeline matched with some of the clusters.

From the gut content, two polychaete *16s rDNA* fragments were successfully amplified. One sequence matched genera of the family Terebellidae (whole sequence, PID~80), such as *Neoamphirite* Hessle, 1917, *Leaena* Malmgren, 1866, or *Polycirrus* Grube, 1850; the second sequence marginally matched genera of the family Spionidae (last 100 bp, PID~80), such as *Spio* Fabricius, 1785 and *Saccocirrus* Bobretzky, 1872.

### 2.2. The Venom Cocktail of Raphitoma purpurea

#### 2.2.1. Post-Translational Modification

The majority of PVC related to toxin modification processes were retrieved from the venom duct, including peptidases and hydrolases of the S10, S9, and M1 families, prolyl-4-hydroxylases (P4H), and peroxiredoxin 4 (PRX4). The S10 serine carboxypeptidase, predicted in the endoplasmic reticulum, was the most highly expressed and matched (PID~34) a venom serine carboxypeptidase from the salivary glands of the Triton’s trumpet *Charonia tritonis* (Linnaeus, 1758) [47]. The S10 family of peptidases cleaves the C-terminal region of peptides and is commonly associated with post-translational modification [48]. Physiological functions related to this enzyme activity include regulation of angiotensin release [48], as well as some neurotoxic properties in bee venom [49]. The M1 peptidase was one of the largest proteins identified. It was located in the endoplasmic reticulum and included an N-terminal catalytic domain associated with aminopeptidase N and Leukotriene hydrolase A4, as well as a C-terminal ERAP1-like domain. This enzyme cleaves the N-terminal residues of oligopeptides and is involved in several processes, including immunity, digestion [50], and toxic activity, such as toxin maturation or prey tissue degradation in snakes [51,52]. An aminopeptidase M was also retrieved from the salivary gland of *Charonia tritonis* [47]. The S9 prolyl endopeptidase was represented by one endoplasmic reticulum isoform, and another was located extracellularly (PID~56). They both matched a prolyl endopeptidase from the digestive secretions of the vampire snail *Cumia reticulata* (Blainville, 1829) [8]. This enzyme cleaves the C-terminal prolyl region of propeptides and is related to toxin maturation in fungi [53]. Two isomers of P4H were retrieved and located extracellularly. P4H is mainly responsible for the post-translational modification of collagen [54] and also works as a chaperone for conotoxins in *Conus* [55,56]. PRX4 had a striking similarity (PID~90) with a peroxiredoxin retrieved from *Profundiconus vaubani* (Röckel and Moolenbeek, 1995) [57]. This family of antioxidant proteins is essential for reducing hydrogen peroxide and other dangerous substances in cells but can also take part in structural modification processes involving cysteine bonds and is commonly found in snake venom glands [58,59].

The only transcript with functions related to venom processing retrieved from the salivary glands was copper type II peptidylglycine α-amidating monooxygenase (PAM). This enzyme is common in all metazoans and performs C-terminal peptide amidation [60], which is also considered one of the most frequently occurring post-translational modifications of conotoxins [61].

Finally, five additional venom processing transcripts were retrieved from all tissue samples and carcases, including two disulfide isomerases, a cyclophilin, and a hydrolase. One of the two disulfide isomerase isomers was retrieved as extracellular, while the other, with lower expression levels, was retrieved as located in the endoplasmic reticulum. They matched disulfide isomerases from the cone snails *Conasprella coriolisi* (Moolenbeek and Richard, 1995) (PID~79) and *Conus magus* Linnaeus, 1758 (PID~70), respectively (additional information in Section 2.3). Disulfide Isomerases are oxidoreductases involved in the folding of proteins by catalysation of disulfide bonds [62] and represent a highly diversified family of toxin chaperones in *Conus* [23,63]. The raphitomid cyclophilin resembled the CeCYP16-like ortholog from *Caenorhabditis elegans* Maupas, 1900, whose function is not yet completely understood [64]. Generally, cyclophilins belong to the peptidyl-prolyl cis-trans isomerases (PPIase) involved in peptide folding, immunosuppression, and anti-parasitic activity [64,65]. Finally, the glycoside hydrolase 47 protein, with a similarity match with *Profundiconus* (PID~81), was found in the salivary glands and carcases. In *Conus*, these hydrolases are hypothesised to enhance the effectiveness of other toxins in venom [66].

#### 2.2.2. Regulation

The transcripts that might be related to regulatory functions in the venom duct were a C-type lectin, two isomers of neprilysin, a GDA1/CD39 nucleoside phosphatase-like protein, and a SUSHI repeat-containing protein. The C-type lectin was the most expressed among these PVCs. C-type lectins are calcium-dependent sugar-binding proteins that act as receptors with a major defensive function in innate and adaptive immunity [67,68]. These proteins were also retrieved in snake venoms, where they can disrupt prey coagulation pathways [69], and in *Profundiconus* [57]. Two isoforms of a neprilysin-like peptide were retrieved (PID~80), one located in the plasma membrane and one truncated and located extracellularly. Neprilysins are M13 membrane metalloproteinases involved in the regulation of peptide signalling [70,71], but they also function as neurotoxins in spiders, snakes, and jellyfishes [72] and can be secreted to cleave the components of the extracellular matrix, facilitating the spreading of other toxins [73]. The GDA1/CD39 nucleoside phosphatase-like protein belongs to a family of extracellular hydrolases that convert ADP to ATP and ATP to ADP and is involved in signalling and regulation of several physiological pathways [74]. The SUSHI repeat protein contains two SUSHI domains, which are commonly found in complement control proteins, adhesion peptides involved in protein-protein or protein-ligand interactions [75,76].

In salivary glands, the transcripts associated with regulation were two elevenins, an EF-hand protein, a hormone, a peptide with two von Willebrand factor A-like (vWFA) domains, and an amyloid A4 precursor. The two elevenins were only slightly similar (PID~35%), and one was more highly expressed and matched the elevenin M2 from *Conus magus* (PID~80). Elevenins are neuropeptides involved in many signalling pathways, including the regulation of salivary secretions. In *Conus*, elevenins are also employed as toxins, mimicking the prey’s neuroactive peptides [77]. EF-hand proteins have high calcium affinity and are involved in most cell communication processes [78]. In snake venom, EF-hand-like toxins have been linked to salivary secretion control [79]. The hormone had a ~60 PID blast similarity with neuropeptide F from *Conus magus* [80], which regulates reproductive and feeding behaviour in invertebrates [81]. The vWFA protein was located in the lysosome and had a similarity match (PID~25) with a protein from the salivary gland of *Cumia reticulata* ([8]). These domains are found in a large number of proteins, like intracellular DNA-repairing enzymes, extracellular collagen or membrane receptors [82]. Von Willebrand factors also play an important role in coagulation and cell adhesion, and vWFA-like toxins are widespread in snake and predator gastropods [8,83,84] or in parasites like *Plasmodium* [85]. The amyloid A4 precursor included heparin and a copper-binding domain. This peptide is responsible for neurite outgrowth and is fundamental in Alzheimer’s disease treatment [86]. Additional physiological functions outside the brain have been observed in the intestinal epithelium and salivary glands of insects [87].

Several transcripts were identified in all tissue samples: two ferritins, a calreticulin, the delta subunit precursor of the translocon-associated protein (TRAP), an exonuclease-endonuclease-phosphatase (EEP), a von Willebrand Factor C peptide, a transposase, a saposin-rich protein, an ML domain protein, two cathepsins and two galactose-binding lectins. Two ferritin transcripts, one cytosolic and one extracellular, were highly expressed in the carcases and salivary glands and moderately expressed in the venom duct. A high similarity match (~91 PID) was retrieved with the *Conus magus* venom ferritin. Ferritins act as iron-storage in cells and are involved in iron transportation when secreted, and they are also involved in the development, regulation, and shell growth in molluscs [88]. The calreticulin was expressed at a medium level in the salivary glands and located in the endoplasmic reticulum. This peptide is fundamental in chaperoning and cell storage of calcium, but venom calreticulins are also known to be involved in invertebrate parasitic interactions, where they inhibit the immune reaction [89,90]. The TRAP-delta subunit was found to be extracellular. In cells, this complex is fundamental for translocating secretory proteins across the plasma reticulum membrane [91]. The EEP domain is shared by a large number of proteins with phosphodiester cleaving capacity, which mostly bind to nucleic acids, like DNA-repairing enzymes, or signal peptides [92]. It is also present in the bacterial cytolethal distending toxins, which damage the DNA [93]. The vWFC-like protein was the largest identified among all PVCs, with an N-terminal vWFC-like domain and a C-terminal collagen-like domain. VWFCs are present in the cartilage matrix and regulate extracellular protein binding [82,94]. Transposases are responsible for moving DNA fragments inside the genome and could be involved in the replacement of defective genes or in gene duplication. They are also very compelling tools for gene therapy [95]. Both ML and saposin domains are lipid-recognition enzymes involved in immunity and lipid catabolism. They can bind to pathogen-related products and trigger their degradation [96,97]. Of the two cathepsin transcripts, one included a cathepsin inhibitor domain and a papain-like cathepsin domain, and the other had two additional cystatin-like domains in the N-terminal region. Cathepsins are cysteine lysosomal endopeptidases of the C1 peptidase family and take part in a broad range of physiological activities, including immunity and anti-toxin activity [98]. These proteins are inactive when translated by cysteine protease inhibitors in propeptide regions, such as cystatins [98]. These transcripts had a blast match with cysteine-rich secretory proteins of *Charonia tritonis* [47]. Galactose-binding lectins are sugar-binding proteins involved in many physiological processes based on a carbohydrate recognition process. They also take part in envenomation in snakes, disrupting the prey haemostasis system [99].

#### 2.2.3. Biosynthesis

Most of the venom duct transcripts were identified as conotoxins due to a match in either their whole sequence or the signal sequence. Three transcripts matched with *Conus litteratus* Linnaeus, 1758 precursor Lt22.2 (PID~65), and were the PVC with the highest similarity with a known conotoxin (additional information in Section 2.3). With the exception of conkunitzins (additional information in Section 2.3), all remaining toxins had no similarity match with known venom products, and only a few were predicted in the conotoxin families A, M, and O1. They were best characterised following additional domains identified in the peptide sequence, including Kunitz/BPTI, secapin, and agatoxin. Two isomers had a single BPTI domain, and four isomers had a double BPTI domain. Kunitz/BPTI proteins are protease inhibitors widespread in venomous organisms, with functions related to haemostasis impairing as thrombin inhibitors and ion-channel modulators [100]. In *Conus*, these toxins are called conkunitzins and act as powerful channel-blocking neurotoxins [101]. The conkunitzins retrieved in *Raphitoma* had a similarity match with both tick species (PID~40) and *Californiconus californicus* (Reeve, 1844) and *Conus ermineus* Born, 1778 (PID~50). Secapin was retrieved in two isomers. This protease inhibitor is a known component of bee venom involved in immunity, anticoagulation, and neurotoxicity [102,103]. Two agatoxin transcripts were retrieved, one more expressed in the venom duct and one in the salivary glands. These powerful spider neurotoxins induce paralysis by affecting synaptic neuromuscular junctions [104]. There were also multiple low-score matches with tick salivary peptides, haemadin, and clavanin. Ticks are parasites that feed on the blood of their hosts and employ anticoagulants to facilitate feeding [105]. Salivary secretions also contain neuroactive toxins that reduce the immune response and induce paralysis to avoid detection and prevent removal [4,106]. Two peptides had an N-terminal clavanin-like and a C-terminal haemadin-like domain. Haemadin is an anticoagulant toxin secreted by leeches [107] with promising pharmacological applications [108], whereas clavanins are antimicrobial peptides from tunicates [109]. The remaining transcripts remained mostly or completely uncharacterised, including transcripts with the following domains: Beta flower 3 and DUF3931 (with the second highest expression level), DUF885, heavy metal binding, macin, defensin, transmembrane 219, and apolipoprotein M.

The salivary gland toxins included conotoxins, a ligand-gated ion channel (LGIC) receptor, a ShK cysteine-rich secretory protein (CRISP), a cardioactive peptide (CAP), and pentraxins. The salivary conotoxin-like peptides could not be identified with any known cone snail family, but two of them were similar (PID~65) to the peptides retrieved in *Hemifusus tuba* Gmelin 1791. The LGIC transcript had a medium expression level and matched the extracellular domain of the nicotinic acetylcholine receptor nAChR2, similar to typical conotoxins of superfamily A [110]. LGIC mediates synaptic transmission and is permeable to ion channels [111]. ShK domains are present in potassium-blocking neurotoxins first isolated from sea anemones [112], and later retrieved in gastropods [8]. The ShK toxin retrieved in *Raphitoma* had ~50 PID, with one from *Cumia reticulata* and ~27 from cnidarians. Cardioactive peptides owe their name to the cardioacceleration function that they are associated with in crustaceans, together with additional regulatory processes. ConoCAP are conid neurotoxins with opposite effects compared to their arthropod counterparts, and their function in venom is still not fully understood [113]. *Raphitoma* conoCAP peptide had a ~55 PID similarity match with a *Conus villepinii* precursor. Two pentraxins were retrieved with a triple N-terminal chitin-binding domain. Pentraxins are pattern-recognition molecules that play an important role in immunity and neural activity in snake venom [114]. Three additional transcripts were retrieved in the salivary glands: a large peptide with a tumour necrosis factor, a peptide with a low-quality match for insulin-like growth factor, and a peptide with a low-quality match for an FMRFamide—also retrieved among wasp neurotoxic components [115].

Finally, several transcripts that were more highly expressed in the carcase samples were retrieved, including conotoxin precursors and conotoxin with Kunitz/BPTI domains, a hormone, a large Kunitz/BPTI and WAP domain protein, and a CRISP protein. Two conotoxin precursors had good similarity matches (PID~50) with the *Conus arenatus* Hwsass 1792 conotoxins of the family DivMKVAVVLLVS, but their expression was outstandingly high in the carcase samples (TMP > 15000), and only low in the salivary glands and venom duct (additional information in Section 2.3). The Kunitz/BPTI conotoxin had BLAST matches with turritoxin (PID~60) from *Gemmula* and *Profundiconus* (additional information in Section 2.3). The hormone matched both the insulin-binding domain and thyroglobulin. The Kunitz/BPTI protein with a triple WAP domain had a similarity match with both the gastropod (PID~30) and the sea anemone (PID~54).

### 2.3. Venom Evolutionary Patterns in Conoidea

The largest number of matches with the homebuilt gastropod dataset was retrieved with *Profundiconus* spp., followed by *Conus magus*, *Charonia tritonis,* and *Hemifusus tuba*, whereas the UniProtKB toxin database had matches mostly with snakes, gastropods, and spiders.

**Figure 3:**
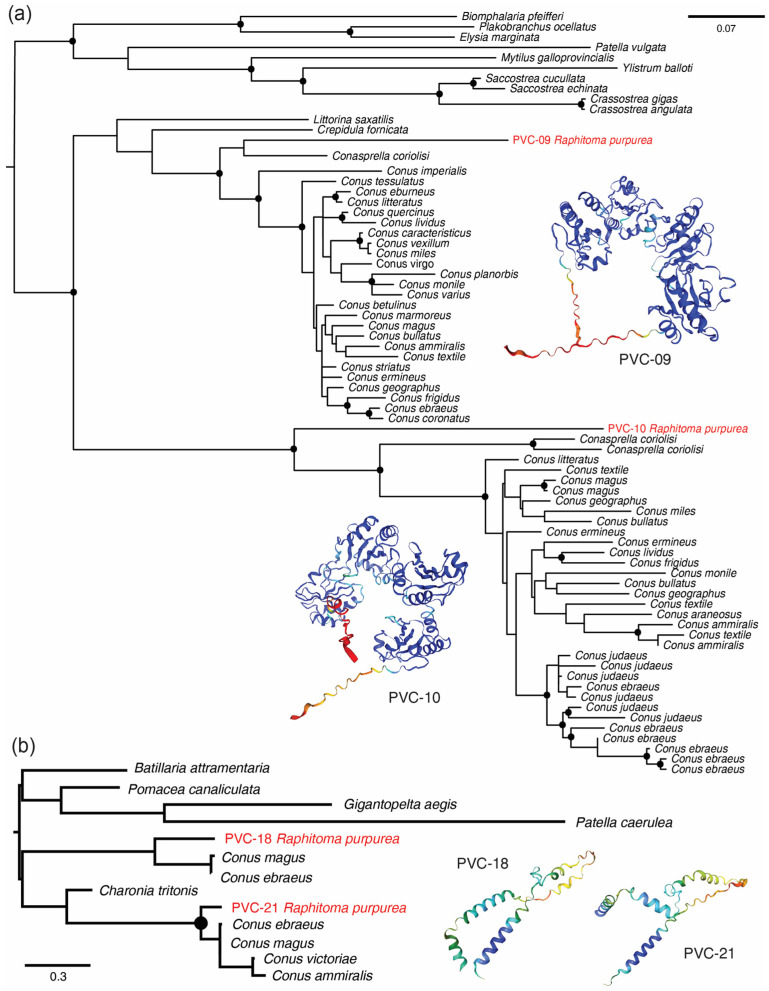
Maximum likelihood relationships of the amino acid sequences of (**a**) disulfide isomerases and (**b**) elevenin from *Raphitoma purpurea* (highlighted in red) with the gastropod sequences from NCBI and UniProtKB. Black dots mark nodes with Ultrafast support > 95%.

**Figure 4:**
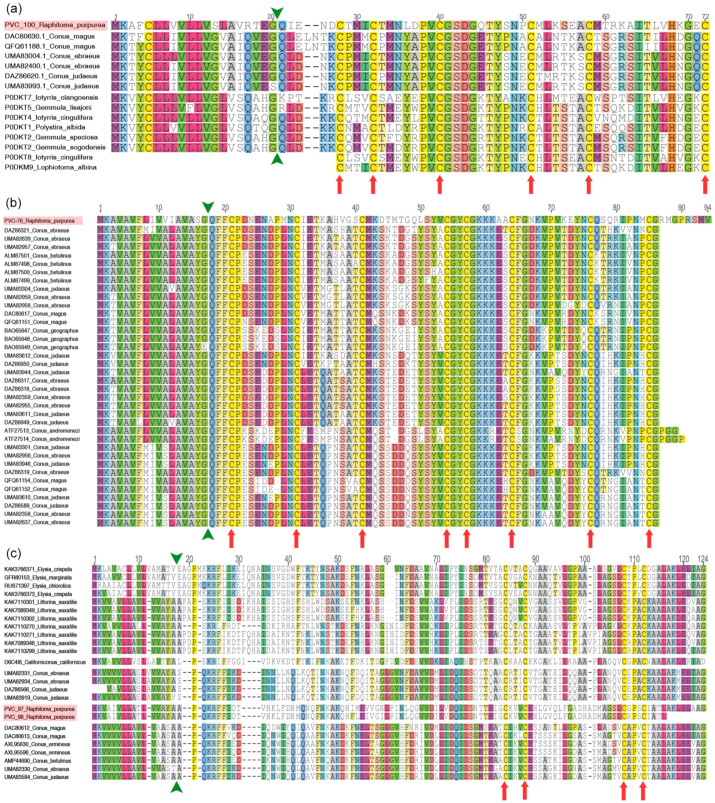
Peptide sequence alignment of (**a**) turritoxin, (**b**) conotoxin Pmag02, and (**c**) conotoxin Cerm06 from *Raphitoma purpurea* (highlighted in red) with gastropod sequences from NCBI and UniProtKB. The green arrow points at the predicted signal sequence cleavage site and the red arrows highlight the cysteine framework.

Remarkable evolutionary results were retrieved from the alignments of the following PVC: the two isomers of disulfide isomerase (PVC-09 and PVC-10), the two isomers of elevenin (PVC-18 and PVC-21), the turritoxin-like peptide (PVC-100), the conotoxin-like precursors from the venom duct and salivary glands (PVC-76, PVC-79, and PVC-81), the conotoxin-like precursors from the carcases (PVC-97 and PVC-98), and the conkunitzins (see below). The two disulfide isomerases (DI) are clustered separately (Figure 3a). Following the results of previous studies on the evolution of DI [23], they correspond to the common gastropod DI and to a venom-related DI family discovered in Conidae, respectively. A similar pattern was observed for the two isomers of elevenin (Figure 3b), although very few conoidean peptides were available. The raphitomid turritoxin-like transcript (Figure 4a) had high similarity in the signal, propeptide, and mature peptide regions with the peptides retrieved in Turridae (genera *Iotyrris*, *Gemmula*, *Lophiotoma*, and *Polystira*) and Conidae (genus *Conus*). The conotoxin-like precursor (Figure 4b) from the salivary glands and the venom duct was successfully aligned with the Pmag02 conotoxins [80], also retrieved from the salivary secretions of *Conus virgo* [116], whose function has not yet been determined. The conotoxin-like precursor retrieved in the carcase samples (Figure 4c) matched with the conotoxin hyaluronidase of superfamily Cerm06 [117], as well as a number of unidentified proteins from the non-conoidean gastropods *Littorina saxatilis* (Olivi, 1792) and *Elysia* spp. Remarkably, the propeptide region of the *Raphitoma* sequences was similar to that of cone snails, whereas the propeptide region of *Californiconus californicus* (Figure 4c), disregarding the closer phylogenetic relationship, was more similar to non-conid sequences.

The only putative raphitoxin families that produced significant alignments were PRF-02 and PRF-03 (Supplementary Figure S1). PRF-03 conkunitzin had a 77 aa long highly conserved N-region (PID~99), which was not retrieved for PRF-02 or any conid sequence. PRF-02 and PRF-03, including PVC-56, PVC-77, PVC-80, and PVC-84, had a double Kunitz/BPTI domain, which matched well (PID~40, including two cysteine residues) with four peptides retrieved in *Conus ermineus* and *C. magus*. Two additional raphitomid conkunitzins, PVC-54 and PVC-69, had a single Kunitz/BPTI domain, and their sequences were significantly shorter than those of the rest of the raphitomid conkunitzins, similar to most of the *Conus* peptides. The remaining PRF did not match with any known conotoxin of the corresponding predicted superfamily or cysteine framework.

## 3. Discussion

Our approach to the transcriptomic data analysis of *Raphitoma purpurea* was rather conservative: we adopted a recently developed pipeline that scores transcripts taking into account sequence and structural homology and expression levels. This allowed us to limit our search to the transcripts more likely involved in envenomation while at the same time focusing not only on toxins but on a broader set of secreted proteins and peptides possibly related to venom functions. Notably, the highest number of matches was retrieved with studies that performed a broader characterisation [8,57,80,118], regardless of the evolutionary relatedness of the studied taxa. During the manual annotation process, several transcripts were discarded, indicating that a manually curated assessment is still recommended to validate the results of the automated pipelines [42,119]. Compared to methods that rely on a single detection strategy or lenient filter parameters, our analysis is less likely to generate false positives; however, there could be a higher number of false negatives, affecting the completeness of the venom cocktail characterisation [42]. Although a stringent approach was adopted to ascertain the function of *R. purpurea* PVC, all toxins described in this study still need to be considered as strictly “putative”, as further functional assessment is always required to corroborate transcriptomic results.

The strictest requirement applied during filtering was the expression level. We assumed that putative venom components (PVC) that provide the highest advantage (e.g., in terms of efficacy, lethality, and fitness) would have the highest expression in the envenomation-related tissues while being absent or lowly expressed in the carcases. This led to the discarding of most of the toxin candidates (~99%). Additionally, some PVCs were also expressed in the carcases, raising questions about the validity of the results. In fact, the transcriptome from carcase samples necessary for tissue-specific expression comparison is not always available in venom profile studies [80,117,118]. The conotoxin-like peptides of the Cerm06 superfamily are an example of a dubious result: in *Raphitoma* they were retrieved mainly from the carcases (TPM > 15,000), and the sequences were similar to unidentified proteins from non-conoidean taxa. It is difficult to determine if transcripts with these characteristics are false positives; the expression in envenomation-related tissues alone does not exclude a metabolic housekeeping function [44]. On the other hand, expression in non-venom-related organs could be explained by the acquisition of a novel function and does not exclude the venomous role of a transcript [90]. Furthermore, the presence of similar peptides in distantly related taxa could be explained by evolutionary convergence or gene neofunctionalisation events. The quantification of expression is helpful in limiting the enquiry to the most relevant venom components, but it cannot prove or disprove a transcript involvement in envenomation. This uncertainty determines the possibility of introducing false positives in toxin reference databases, affecting the results of future venom assessment studies and highlighting the need for bioactive assays to confirm transcriptomic study results.

Among the thousands of transcripts initially identified as candidate putative toxins, only 353 had a good BLAST match (PID > 60) with the reference toxin databases. After filtering, only 14 of them were retained as PVC. Therefore, most of the putative toxins retrieved, including ~80% of venom duct toxins, lacked a good similarity match and were retained solely for their expression level and structural features. The scarce number of matches in the *Raphitoma purpurea* secretome revealed a set of venom components completely new to science, including a large number of conotoxin-like peptides, raphitoxins, and several regulation and maturation factors. Assessing more species is necessary to understand how raphitomid venom complexity influenced the evolution of this family. This result also highlights an issue with several transcriptomic analysis steps, including filtering and annotation, which rely on reference databases and are therefore biassed toward the more studied venomous groups (e.g., snakes). Large-scale venom characterisation, especially for gastropods, is lacking, and transcriptomic studies are often focused on specific research questions (e.g., the retrieval of conotoxins). As such, a large number of secretome peptides are never uploaded in the reference databases or provided as Supplementary Data with the publication and remain available only in the raw sequencing data, requiring great effort for comparative analyses. Ironically, this study revealed that this overlooked portion of the venom secretome could be most interesting from a macroevolutionary perspective (see below).

The analyses of the secretome of salivary glands and venom duct of *Raphitoma purpurea* revealed a cocktail of peptides and proteins, including maturation enzymes, regulation factors, and toxins, some of which are similar to those retrieved in the venoms of gastropods, snakes, scorpions, spiders, bees, ticks, leeches, and cnidarians. Most of the putative venom components retrieved were short conotoxin-like peptides, for which 20 different families were identified based on the signal sequence. The software for conotoxin identification failed to predict the superfamily for many raphitoxins, reflecting their diversity from conotoxins. Most of the successful predictions were with the conotoxin superfamilies A, O1, M, and conkunitzin. Four putative raphitoxin families (PRF) showed signs of expansion, including those with the highest expression in the salivary glands (PRF-04, *n* = 23) and in the venom ducts (PRF-01, *n* = 20; PRF-07, *n* = 16; PRF-08, *n* = 10). The high number of duplication events could underlie the process of adaptive evolution, which may be linked to an expansion of the trophic niche [120]. Considering that the radiation in Conidae is believed to be linked to the great diversification of their toxins [121,122], the exceptional diversity in Raphitomidae in terms of species richness, morphology, anatomy, and distribution could disclose an even more surprising toxin diversity.

The molecular identification of polychaetes in the gut content of *R. purpurea* (NCBI accession number: PQ149932–PQ149934) confirmed a vermivore diet for the genus, in agreement with the retrieval of *Polycirrus* sp. remains in the gut of *R. bicolor* (NCBI accession number: PP913530), but the low identity scores of the *R. purpurea* gut barcodes do not allow precise identification of the target prey species or genera. In addition, the primers adopted were specific to polychaetes; therefore, the possibility of a generalist diet cannot be excluded. The toxins retrieved in *Raphitoma* are linked to predatory behaviour: they might inhibit muscular and neural response [110] through neural receptor impairing (conotoxin A, conotoxin M, nAChR antagonist, neuropeptide F, EF-hand proteins, agatoxins), or ion-channel clocking (conotoxin O1, conkunitzins, ShK proteins), and disrupt the prey’s defensive mechanisms (hydrolases, peptidase inhibitors). The conotoxins that matched the *Raphitoma* PVC were all produced by worm-hunting *Conus* species [122,123], and the conotoxin families A, M, O1, and conkunitzin identified among the PRF were also all retrieved in vermivorous cone snails [5]. This result could corroborate a vermivorous diet for Raphitomidae, although toxin profiles do not directly correlate with trophic ecology [5]. Although the composition of the venom of *R. purpurea* could be an adaptation to predation, we cannot exclude a role for adaptive processes related to defence or competition. In order to answer these ecological questions, more species of Raphitomidae should be assessed from both a venomous and a dietary perspective.

In cone snails, venom secretions in the proximal and distal portions of the venom duct can differ at the ecological (defensive vs. predatory response), histological (secretion vs. transport tissue), or toxin maturation levels [124]. This has been corroborated in many species by transcriptomic and proteomic data [125,126,127], whereas some species do not exhibit venom duct compartmentalisation [128]. Our study did not retrieve a pattern of differential toxin secretion between different regions of the venom duct, with the exception of a higher number of proteins involved in muscular contraction retrieved in the proximal portion, reflecting a functional affinity of that specific region with the contiguous venom bulb, whose muscular action pushes the venom towards the foregut. This result suggests that differential secretion of the proximal and distal venom ducts could be an apomorphic trait of cone snails. However, our analysis also pinpointed a certain degree of interindividual variation in venom cocktail composition, which calls for an extension to a larger number of samples to confirm this hypothesis.

The venom duct contained the largest number of PVC, but many were also retrieved from the salivary glands, including conotoxins. The involvement of salivary gland secretions in the envenomation process is discussed in [116]. While the toxins exclusively expressed in the venom duct of *R. purpurea* had high expression levels and did not match with any known venom toxins, the ones also expressed in the salivary glands had lower expression levels and were similar to other conoidean toxins. This set of toxins might thus represent early adaptations towards toxicity in Conoidea. The gene expansion of the conotoxin folding disulfide isomerases was fundamental for the evolution of cone snails’ venom [23], and the results of this study show that this process may have originated before the radiation of Conidae. Similarly, the two elevenins retrieved could be the result of a duplication event of a regulatory factor followed by its neofunctionalisation as a toxin, although only a small number of gastropod elevenins are available in reference databases to adequately support this claim. Furthermore, turritoxins, Pmag02 conotoxins, and conkunitzins in *Raphitoma* could also represent ancestral adaptations of the conoidean venom. If this hypothesis is correct—and excluding a secondary loss of functions—most conoideans families could share these traits [31]. Alternatively, the presence of these venom components in *Raphitoma* would represent instances of convergent evolution.

Overall, our results might indicate that ancestral venom elements were originally produced in the salivary glands, possibly before the development of the venom duct. Subsequently, these gene families expanded following the evolution of the venom duct, where a larger number of toxins could be secreted at a higher concentration. The histological origin of the venom duct is the mid-oesophageal gland, while the salivary glands are strictly related to the radular sac [122,124,129], representing a more isolated, safe environment for the secretion of toxins. This would suggest that at least part of the venom evolution may have started in the salivary glands, where early venom components were employed. If this hypothesis is correct, evidence of venom evolution in Conoidea is more likely to be found in toxin maturation and regulation factors and venom components of the salivary glands rather than in venom duct conotoxin-like peptides, which probably had their evolutionary burst during the radiation of the family Conidae.

## 4. Materials and Methods

### 4.1. Dataset

Fifteen specimens of *Raphitoma purpurea* were collected in Pointe de l’Arcouest, Ploubazlanec (Brittany, France) from 24 May to 16 June 2023. Upon collection, each shell was cracked, specimens were fixated in cold NucleoProtect^®^ RNA (Macherey-Nagel, Düren, Gernamy), and kept at room temperature (3 to 12 days) before storage at 4 °C. Shortly after, the salivary glands and the venom duct were dissected on ice, dried with paper, snapped into small pieces, and dissolved in TRI Reagent™ solution (ThermoFisher Scientific, Waltham, MA, USA). The entire digestive tract was also dissected and preserved in ethanol. Eleven carcases, three whole bodies, six salivary glands, and twelve venom ducts were successfully processed for transcriptome analyses. Two of the venom ducts were dissected into their most proximal and distal sections, removing the middle portion to be processed separately. RNA extraction, quality check, and sequencing were performed by BGI (Hong Kong, China). Taxonomic identification of all samples was confirmed by *cox1* amplification from a tissue clip of the foot.

After assessment of the concentration and quality of extracted RNA, three salivary glands (SG), three whole venom ducts (VD), two distal fragments of venom duct (dVD), and two proximal fragments of venom ducts (pVD) were sequenced using DNBSEQ technology in 100 Paired-End with the Smart-Seq2 kit (BGI Genomics, Hong Kong, China). The three carcases that yielded higher concentration values were sequenced in 150PE with the Poly-A kit (Table 2).

### 4.2. Bioinformatic Analyses

All bioinformatics analyses were performed on the Terastat2 supercomputing cluster of Sapienza University of Rome [130]. Raw reads were trimmed and assembled using Trinity v2.8.6 [131] with a 31 k-mer size and the following TRIMMOMATIC parameters for quality trimming: “SLIDINGWINDOW:4:15 MINLEN:36 LEADING:3 TRAILING:3”. The abundance of transcripts was estimated using the RSEM method using bowtie2 and normalised using the TMM method [132]. Differential expression analysis among different tissues was performed using DESeq2 [133] with the *p*-value set to 0.01.

A reference dataset was built, including all toxins from UniprotKB server (https://www.uniprot.org search keyword: KW-0800, accessed on 22 January 2024) and Conoserver (https://www.conoserver.org, accessed on 22 January 2024), and the published toxins of 33 gastropod species of the genera *Charonia* Gistel, 1847, *Conasprella* Thiele, 1929, *Conus* Linnaeus, 1758, *Cumia* Bivona, 1838, *Hemifusus* Swainson, 1840, *Profundiconus* Kuroda, 1956, *Pygmaeconus* Puillandre and Tenorio, 2017, *Purpuraturris* K. Chase, Watkins, Safavi-Hemami and B. M. Olivera, 2022, and *Turris* Batsch, 1789 [5,8,47,57,80,117,118,119,122,134,135,136,137,138].

Candidate venom components were identified using DeTox (https://github.com/Hyperdiverseproject/DeTox, accessed on 12 March 2024), a user-friendly pipeline that combines several software for sequencing data preprocessing and toxins detection [42]. All parameters were set to default. In this pipeline, transcripts were translated using orfipy v0.0.4 [139] and clustered with CD-HIT v4.8.1 [140] to reduce redundancy. Then, putative toxins of at least 35 amino acids were scored considering four features: (i) peptide sequence similarity with the reference database (B) using diamond v2.1.8 [141], (ii) detection of functional domains against the Pfam database (D) using HMMER v3.3.2, (iii) detection of a signal sequence and no transmembrane domain (S)—features typical of secretion components—using SignalP 5.0b [142] and Phobius v1.01 [143,144], and (iv) presence of a cysteine framework (C). The output contained all transcripts with at least one positive score. WoLF PSORT was also implemented to predict the subcellular localisation [145].

In order to reduce the number of non-venom-related genes and contaminations, a BUSCO [146] search of the transcriptome was conducted with several reference databases (Metazoa, Eukaryota, Bacteria, Archaea, Fungi, and Mollusca). All matching transcripts were filtered from the DeTox output.

### 4.3. Venom Cocktail Characterisation

Two subsets were extracted from the DeTox output: the first included the most relevant putative venom components (PVCs) based on the DeTox score and expression levels, and the second comprised potential conotoxin-like families in *Raphitoma* (raphitoxins) referred to as putative raphitoxin families (PRFs) retrieved using ConoDictor v2.3.5 [147] and a modified pipeline from [116] (see below).

PVCs were selected based on the following criteria: at least 10 TPM in the salivary glands or venom duct, a positive DeTox score for reference match (B or D), and toxin-like structural features (S or C). Additionally, all peptides with high expression levels (>1000 TPM) were retained, regardless of their DeTox scores. All surviving transcripts were annotated integrating all the information derived from HMMER v3.3.2 [148] with the Pfam database and the e-value cut-off set at 0.01, eggNOG Mapper 2.1.12 [149,150] run on the GalaxyEU server (https://usegalaxy.eu, accessed on 22 March 2024), HHpred (https://toolkit.tuebingen.mpg.de/tools/hhpred, accessed on 4 April 2024) with the Pfam, NCBI CD v3.19, SMART v6.0, and proteasome homologs Jun 21 databases [151,152,153], UniProtKB align tool (https://www.uniprot.org/align, accessed on 4 April 2024) and BLAST of both peptide and nucleotide sequence against the non-redundant (nr) database [154,155]. The 3D structure of PVCs was predicted using alphafold2 on the GalaxyEU server [156,157] or ColabFold v1.5.5 [158]. During the annotation process, the level of expression in the carcase samples was taken into account and compared with the expression in the salivary glands or venom duct. Given the possibility of intraspecific (or even intraindividual) variation in venom composition [4,43] and the reduced number of replicates in our dataset, the highest TPM among samples in each tissue was taken into consideration instead of the average. The expression levels were defined as low (TPM > 10), medium (TPM > 100), and high (TPM > 1000). All annotated PVC are listed in Section 2.1 (Table 1).

In order to retrieve conotoxin-like peptide families, the candidate venom components were filtered using ConoDictor v2.3.5 [147] and clustered by their signal sequence using CD-HIT v4.8.1 [140] with a 0.65 threshold. Clusters with fewer than three transcripts, long stretches of amino acid repeats in the sequence, less than 10 TPM, and mature regions < 20 aa were discarded. All retained transcripts were checked using BLAST against the non-redundant (nr) database to ensure no match with non-toxic components. The remaining clusters constituted the PRF, which were separately aligned using MAFFT (https://mafft.cbrc.jp/alignment/server/, accessed on 29 May 2024) with the G-ins-l iterative refinement method and 0.2 align level. Alignments were checked and manually edited using Geneious Pro 4.8.5. The peptide regions, cysteine framework, conotoxin class, and superfamily of each transcript were retrieved from the ConoPrec tool (https://www.conoserver.org/?page=conoprec, accessed on 29 May 2024; [159,160], and ConoDictor v2.3.5 [147]. Each PRF was assigned to a conotoxin superfamily following the attribution of the majority of transcripts. Previously retrieved PVCs were assigned to the retrieved PRF using CD-HIT v4.8 with a 0.65 threshold on the signal sequence.

### 4.4. Venom Evolutionary Patterns in Conoidea

The number of similarity matches (PID > 60) in the *Raphitoma purpurea* secretome (DeTox raw output) with the homebuilt dataset and the UniProtKB toxin dataset was investigated. PVCs with good BLAST results were aligned with similar gastropod sequences retrieved from NCBI and UniProtKB, with a focus on transcripts showing hints of duplication (e.g., isomers with differential expression in tissues). Each PRF was aligned with conotoxins from the same predicted superfamily or shared the same cysteine framework. Maximum Likelihood trees were generated on IQTree (http://iqtree.cibiv.univie.ac.at/, accessed on 12 June 2024) [161] using the amino acid alignments with an automatic substitution model and 1000 Ultrafast bootstrap. Trees were modified in FigTree 1.4.4 [162] and Adobe Illustrator 1.0. Node values were considered supported when higher than 95.

## Figures and Tables

**Table 1:** List of putative venom components in *Raphitoma purpurea*. Colours represent maximum TPM among samples (white: 0 to 10; blue: 10 to 50; green: 50 to 100; yellow: 100 to 250; orange: 250 to 500; light red: 500 to 1000; dark red: 1000 to 5000; black: over 5000); transcript marked with “x” were differentially expressed in VD or SG, those marked with “!” had expression in carcases within 10 folds of envenomation organs; Matching domains in HHpred with low scores single (~75 p) or double (~50 p) underlined; number of domains specified in parentheses; DeTox rating (see Material and Methods).

ID	C	SG	VD	pVD	dVD	len	Matching Domains	Rating
Post-translational modification								
PVC-01						488	Peptidase S10 serine carboxypeptidase	SBCD
PVC-02			x			943	Aminopeptidase M1, ERAP1	SCD
PVC-03			x			725	Peptidase S9 prolyl oligopeptidase	SBD
PVC-04			x			716	Peptidase S9 prolyl oligopeptidase	SBCD
PVC-05			x			573	Prolyl-4-hydroxylase, tetratricopeptide	SBCD
PVC-06			x			564	Prolyl-4-hydroxylase, tetratricopeptide	SBCD
PVC-07						253	Peroxiredoxin IV	SBCD
PVC-08						445	Copper type II PAM monooxygenase (2)	SCD
PVC-09	!					500	Disulfide isomerase	SBCD
PVC-10	!					492	Disulfide isomerase	SBCD
PVC-11	!					211	Cyclophilin CeCYP16	SBD
PVC-12	!					353	Glycoside hydrolase 47	SBD
Regulation								
PVC-13			x			148	Attractin-like C-lectin	SCD
PVC-14			x			136	Peptidase M13 neprilysin	SCD
PVC-15			x			369	Peptidase M13 neprilysin	SCD
PVC-16			x			446	GDA1/CD39 nucleoside phosphatase	SCD
PVC-17			x			197	SUSHI repeat (2)	SCD
PVC-18		x				113	Elevenin	SB
PVC-19						214	EF-hand protein	SD
PVC-20						81	Neuropeptide F	SBD
PVC-21						122	Elevenin	SB
PVC-22						436	vWFA-like (2)	SBCD
PVC-23						263	Heparin, amyloid A4 precursor	SCD
PVC-24	!					90	Ferritin	BD
PVC-25	!					111	Ferritin	BD
PVC-26	!					414	Calreticulin	SD
PVC-27	!					172	TRAP-delta	SD
PVC-28	!					596	Exonuclease-Endonuclease-Phosphatase	SCD
PVC-29	!					1407	vWFC-like, collagen	SCD
PVC-30	!					165	Transposase, PAX domain	SCD
PVC-31	!					155	ML domain	SCD
PVC-32	!					598	Saposin (7)	SCD
PVC-33	!					560	Peptidases C1 cathepsin, cathepsin inhibitor, cystatin (2)	SBCD
PVC-34	!					351	Peptidases C1 cathepsin, cathepsin inhibitor	SBCD
PVC-35	!					291	Galactose-binding lectin (2)	SCD
Biosynthesis								
PVC-36			x			56	-	S
PVC-37			x			78	Beta flower 3, DUF3931	S
PVC-38			x			65	DUF3530	S
PVC-39			x			71	tick salivary peptide	S
PVC-40			x			91	tick salivary peptide	SC
PVC-41			x			67	Conotoxin, DUF2756, Clavanin, haemadin	SC
PVC-42						94	Conotoxin, spider neurotoxin	SC
PVC-43			x			93	Conotoxin	SC
PVC-44			x			92	tick salivary peptide	SC
PVC-45			x			55	Conotoxin	S
PVC-46			x			80	Conotoxin, Clavanin, haemadin	SC
PVC-47			x			51	-	S
PVC-48			x			78	Conotoxin, secapin, haemadin	SC
PVC-49			x			71	Conotoxin, heavy metal binding	SC
PVC-50			x			48	-	S
PVC-51			x			36	Defensin propeptide	S
PVC-52						72	Conotoxin	SC
PVC-53			x			95	tick salivary peptide, Defensin	SC
PVC-54			x			136	Kunitz/BPTI	SCD
PVC-55			x			105	DUF3931	SC
PVC-56			x			143	Kunitz/BPTI (2)	SBCD
PVC-57			x			74	Secapin	SC
PVC-58			x			38	-	S
PVC-59			x			47	tick salivary peptide	S
PVC-60						88	Conotoxin	SC
PVC-61			x			53	-	S
PVC-62			x			45	-	SC
PVC-63			x			45	-	SC
PVC-64			x			46	Transmembrane 219	S
PVC-65			x			69	Conotoxin, tick salivary peptide	SC
PVC-66			x			81	-	S
PVC-67			x			187	Apolipoprotein M	SBC
PVC-68			x			88	Conotoxin	SC
PVC-69			x			154	Kunitz/BPTI	SCD
PVC-70			x			69	tick salivary peptide	SC
PVC-71			x			90	tick salivary peptide	SC
PVC-72			x			64	-	S
PVC-73			x			110	Conotoxin, macin	SC
PVC-74			x			47	Beta flower 3, DU3931	S
PVC-75			x			36	-	S
PVC-76						94	Myticin preproprotein	SBC
PVC-77			x			232	Kunitz/BPTI (2)	BD
PVC-78			x			55	-	S
PVC-79						41	Pleurocidin antimicrobial 12	SB
PVC-80			x			157	Kunitz/BPTI (2)	SBCD
PVC-81						41	Pleurocidin antimicrobial 12	SB
PVC-82						100	Conotoxin, spider neurotoxin	SCD
PVC-83			x			606	DUF885, nucleoporin FG2, tyrosine carboxypeptidase	SD
PVC-84						143	Kunitz/BPTI (2)	SBCD
PVC-85		x				235	Nicotinic acetylcholine receptor	SCD
PVC-86						115	Conotoxin, chitin-binding	SCD
PVC-87						97	Conotoxin, chitin-binding	SB
PVC-88						120	Chitin-binding	SB
PVC-89						457	CRISP, ShK toxin (3)	SBCD
PVC-90						99	Conotoxin	SB
PVC-91						920	Tumour necrosis factor	SCD
PVC-92						311	Cardio active peptide (2)	SBC
PVC-93						434	Chitin-binding (3), pentraxin	SCD
PVC-94		x				203	Insulin-like growth factor, conotoxin	SCD
PVC-95						391	FMRFamide	SD
PVC-96						417	Chitin-binding (3), pentraxin	SCD
PVC-97	!					116	Conotoxin precursor	SBC
PVC-98	!					108	Conotoxin precursor	SBC
PVC-99	!					86	-	SC
PVC-100	!					70	Conotoxin, Kunitz/BPTI	SBCD
PVC-101	!					185	Insulin-homologue binding site, Thyroglobulin	SCD
PVC-102	!					409	Kunitz/BPTI, WAP (3)	SBCD
PVC-103	!					219	CRISP	SBCD
PVC-104	!					139	Conotoxin, Kunitz/BPTI, Kunitz/BPTI	SCD

**Table 2:** List of carcases (C), salivary glands (SG), whole venom duct (VD), distal venom duct (dVD), and proximal venom duct (pVD) processed for NGS transcriptome sequencing and gut contents Sanger sequencing (GC).

Sample ID	Sampling Data	Date	C	SG	VD	pVD	dVD	GC
BAU 4602	France, Brittany, Ploubazlanec, Pointe de l’Arcouest 48.8209, −3.0100	1 June			x			
BAU 4604		1 June	x		x			
BAU 4605		1 June						x
BAU 4607		6 June	x	x		x	x	
BAU 4609		14 June		x	x			
BAU 4611		16 June	x	x		x	x	x

## Data Availability

Raw sequencing data were deposited in the NCBI under project PRJNA1143428. Detox output containing 74,520 putative toxin candidates is available in Table S1. The 104 putative venom components and 20 raphitoxin families are available in Tables S2 and S3.

## Supplementary Materials

The following supporting information can be downloaded at: https://www.mdpi.com/article/10.3390/toxins16080348/s1, Table S1: Detox output; Table S2: list of PVC; Table S3: list of PRF; Figure S1: Conkunitzin and PRF alignment.
