# Supplementary material for: Potential Ancestral Conoidean Toxins in the Venom Cocktail of the Carnivorous Snail Raphitoma purpurea (Montagu, 1803) (Neogastropoda: Raphitomidae)
Source: Toxins (Basel). 2024 Aug 9;16(8):348. doi: 10.3390/toxins16080348 (PMC11359391; doi:10.3390/toxins16080348)
Supplement: Supplementary file 1 [file toxins-16-00348-s001.zip › Suppl_4_Conkunitzins_and_PRF_alignment.pdf]

Supplementary material 4. Alignment of *Raphitoma purpurea* conkunitzins and PRF. Conkunitzins aligned with with gastropod sequences from NCBI and UniProtKB and trimmed.

conkunitzins

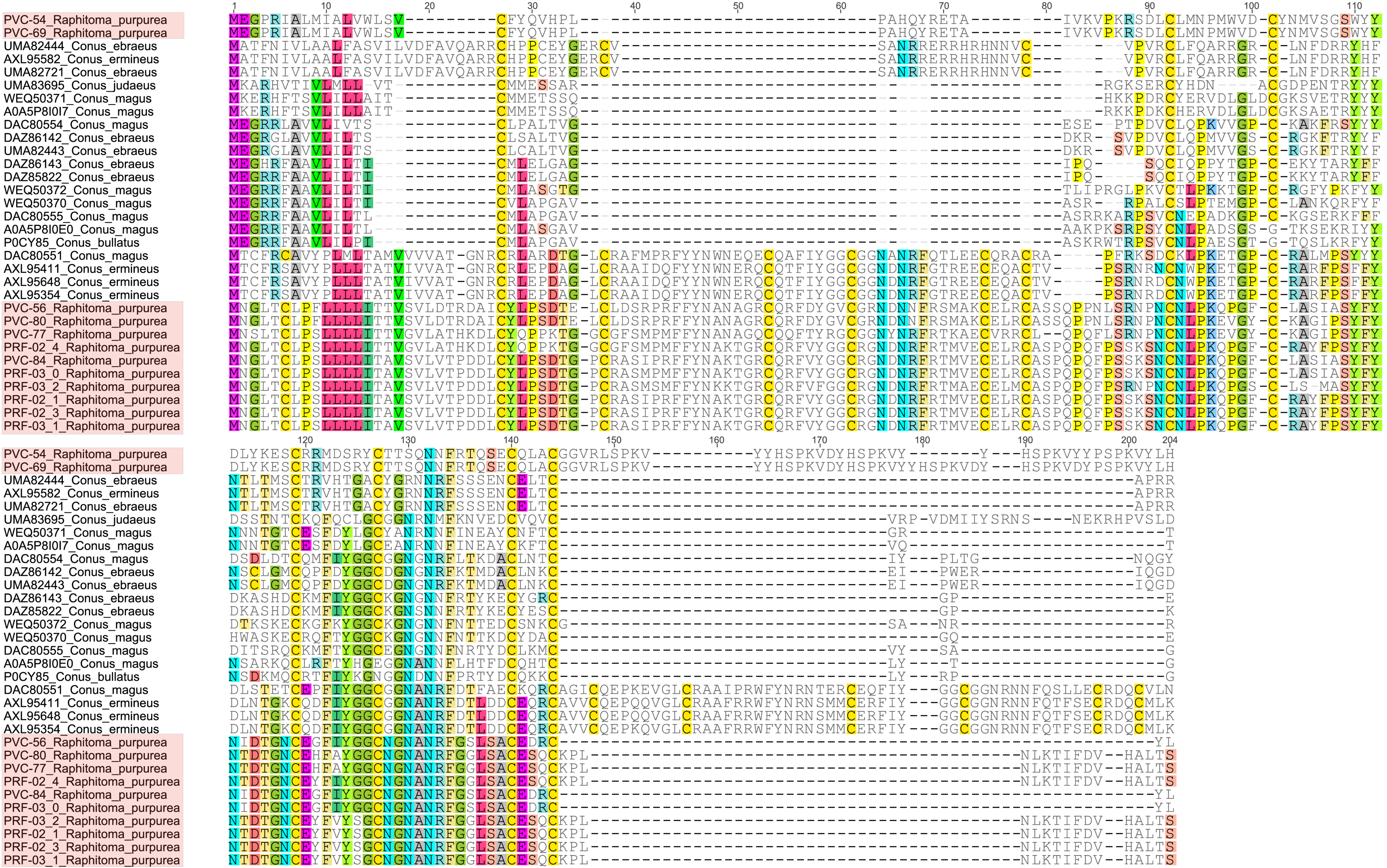

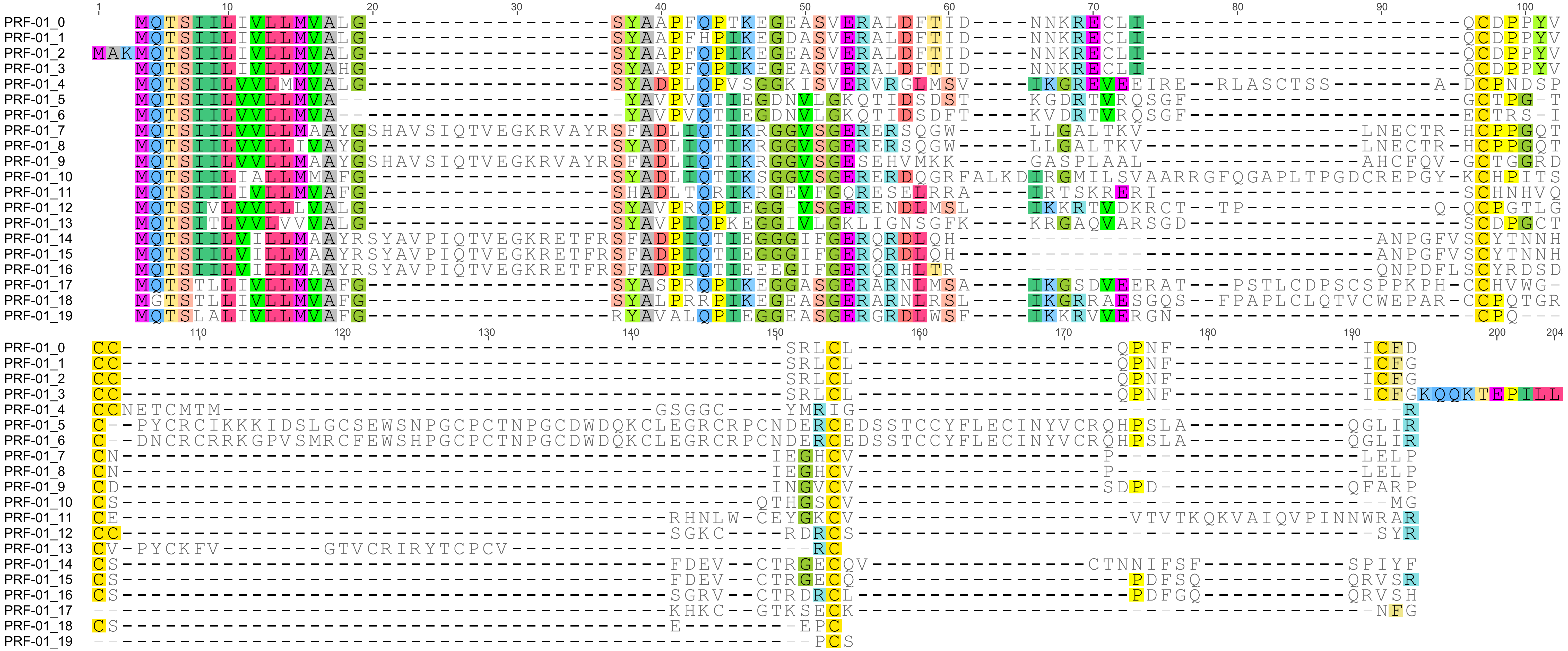

PRF-01_0
PRF-01_1
PRF-01_2
PRF-01_3
PRF-01_4
PRF-01_5
PRF-01_6
PRF-01_7
PRF-01_8
PRF-01_9
PRF-01_10
PRF-01_11
PRF-01_12
PRF-01_13
PRF-01_14
PRF-01_15
PRF-01_16
PRF-01_17
PRF-01_18
PRF-01_19
1
10
20
30
40
50
60
70
80
90
100
110
120
130
140
150
160
170
180
190
200
204

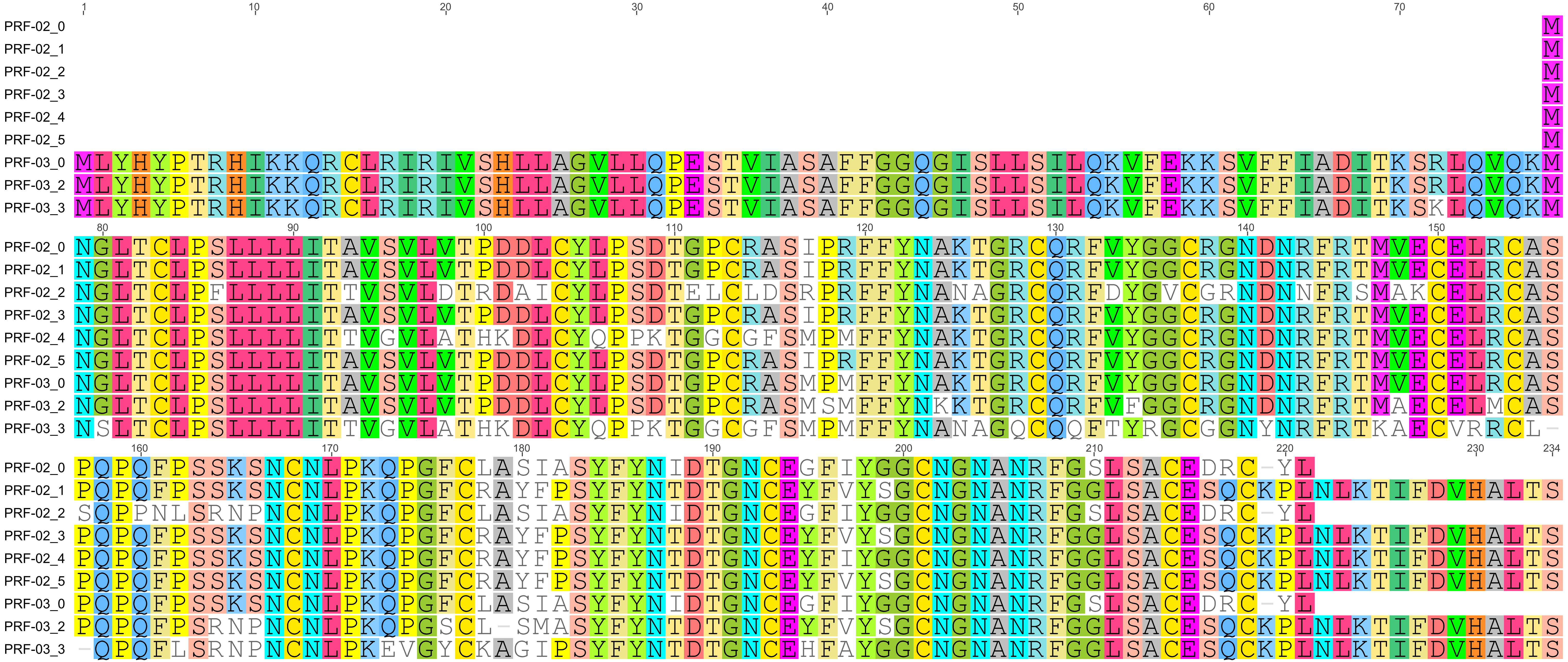
PRF-02_0
PRF-02_1
PRF-02_2
PRF-02_3
PRF-02_4
PRF-02_5
PRF-03_0
PRF-03_2
PRF-03_3

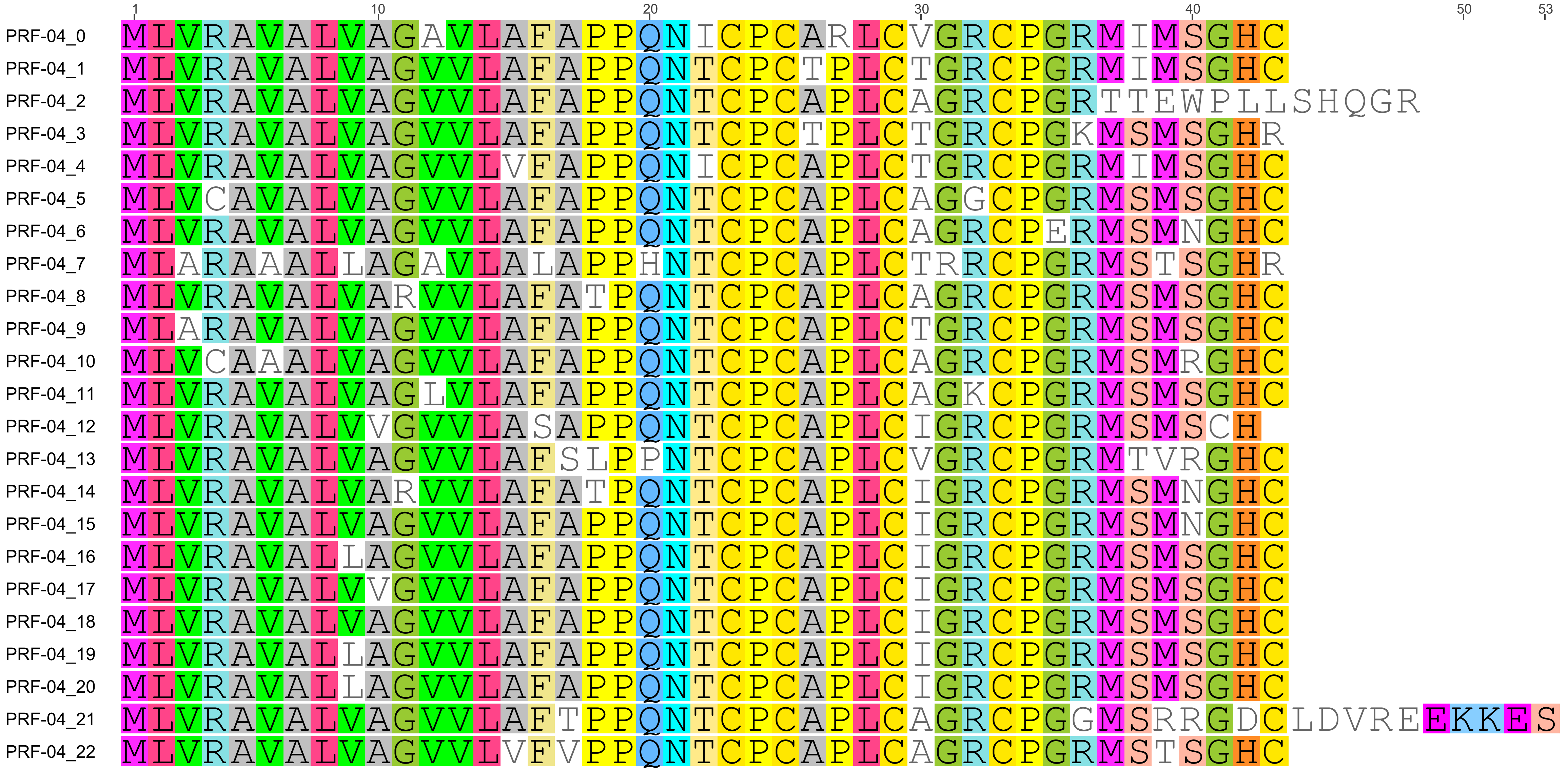
1
10
20
30
40
50
53
PRF-04_0 MLVRAVALVAGAVLAFAPPQNICPCARLCVGRCPGRMIMSGHC
PRF-04_1 MLVRAVALVAGVVLAFAPPQNTCPCTPLCTGRCPGRMIMSGHC
PRF-04_2 MLVRAVALVAGVVLAFAPPQNTCPCAPLCAGRCPGRTTEWPLLSHQGR
PRF-04_3 MLVRAVALVAGVVLAFAPPQNTCPCTPLCTGRCPGKMSMSGHR
PRF-04_4 MLVRAVALVAGVVLVFAPPQNICPCAPLCTGRCPGRMIMSGHC
PRF-04_5 MLVCAVALVAGVVLAFAPPQNTCPCAPLCAGGCPGRMSMSGHC
PRF-04_6 MLVRAVALVAGVVLAFAPPQNTCPCAPLCAGRCPERMSMNGHC
PRF-04_7 MLARAAALLAGAVLALAPPHNTCPCAPLCTRRCPGRMSTSGHR
PRF-04_8 MLVRAVALVARVVLAFATPQNTCPCAPLCAGRCPGRMSMSGHC
PRF-04_9 MLARAVALVAGVVLAFAPPQNTCPCAPLCTGRCPGRMSMSGHC
PRF-04_10 MLVCAAALVAGVVLAFAPPQNTCPCAPLCAGRCPGRMSMRGHC
PRF-04_11 MLVRAVALVAGLVLAFAPPQNTCPCAPLCAGKCPGRMSMSGHC
PRF-04_12 MLVRAVALVVGVVLASAPPQNTCPCAPLCIGRCPGRMSMSCH
PRF-04_13 MLVRAVALVAGVVLAFSLPPNTCPCAPLCVGRCPGRMTVRGHC
PRF-04_14 MLVRAVALVARVVLAFATPQNTCPCAPLCIGRCPGRMSMNGHC
PRF-04_15 MLVRAVALVAGVVLAFAPPQNTCPCAPLCIGRCPGRMSMNGHC
PRF-04_16 MLVRAVALLAGVVLAFAPPQNTCPCAPLCIGRCPGRMSMSGHC
PRF-04_17 MLVRAVALVVGVVLAFAPPQNTCPCAPLCIGRCPGRMSMSGHC
PRF-04_18 MLVRAVALVAGVVLAFAPPQNTCPCAPLCIGRCPGRMSMSGHC
PRF-04_19 MLVRAVALLAGVVLAFAPPQNTCPCAPLCIGRCPGRMSMSGHC
PRF-04_20 MLVRAVALLAGVVLAFAPPQNTCPCAPLCIGRCPGRMSMSGHC
PRF-04_21 MLVRAVALVAGVVLAFTPPQNTCPCAPLCAGRCPGGMSRRGDCLDVREEKKES
PRF-04_22 MLVRAVALVAGVVLVFVPPQNTCPCAPLCAGRCPGRMSTSGHC

```
         1        10        20        30        40        50        60        70        80        90        100       110       120
RPF-05_0 MTALTLVATLICVFAVLCSGRSVFKRTGEEGPFPERECPPGSPMAMCLKVPCLDGAPRPTCQANIPGAHCINNFCGVCKREWDDAKGNNITLACEGIEAVEEELSLFLGSD-------HT
RPF-05_1 MTALTLVATLICVLAVLCSGRSVFKHTSETIRFKERVCPLFAPKALCSTAPCQEGNSRPTCKANIPGAVCIHNFCGACKREWDDANGNNVTLACEGFLAKVIEHTHTAQTSRPNTTHTHT
RPF-05_2 MTALTLVATLICVLAVLCSGRSVFKHTSETIRFKERVCPLFAPKALCSTAPCQEGNSRPTCKANIPGAVCM
RPF-05_3 MTALTLVATLICVFAVLCSGRSVFKQTSERGRYPERVCPPLVPTAICSTAPCLDGNPRPTCKANIPGARCINNFCGACKRAWDDAKGNDVTLACEGFPAKVIDFDDLWH
```

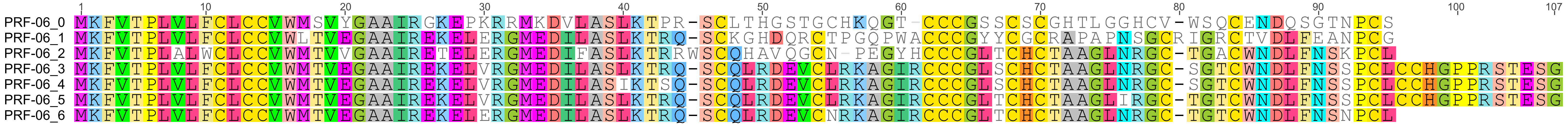
1 10 20 30 40 50 60 70 80 90 100 107
PRF-06_0 MKFVTPLVLFCLCCVWMSVYGAAIRGKEPKRRMKDVLASLKTPR-SCLTHGSTGCHKQGT-CCCGSSCSCGHTLGGHCV-WSQCENDQSGTNPCS
PRF-06_1 MKFVTPLVLFCLCCVWLTVEGAAIREKELERGMEDILASLKTRQ-SCKGHDQRCTPGQPWACCCGYYCGCRAPAPNSGCRIGRCTVDLFEANPCG
PRF-06_2 MKFVTPLALWCLCCVWMTVVGAAIRETELERGMEDIFASLKTRRWSCQHAVQGCN-PEGYHCCCGLTCHCTAAGLNRGC-TGACWNDLFNSKPCL
PRF-06_3 MKFVTPLVLFCLCCVWMTVEGAAIREKELVRGMEDILASLKTRQ-SCQLRDEVCLRKAGIRCCCGLSCHCTAAGLNRGC-SGTCWNDLFNSSPCLCCHGPPRSTESG
PRF-06_4 MKFVTPLVLFCLCCVWMTVEGAAIREKELVRGMEDILASIKTSQ-SCQLRDEVCLRKAGIRCCCGLSCHCTAAGLNRGC-SGTCWNDLFNSSPCLCCHGPPRSTESG
PRF-06_5 MKFVTPLVLFCLCCVWMTVEGAAIREKELVRGMEDILASLKTRQ-SCQLRDEVCLRKAGIRCCCGLTCHCTAAGLIRGC-TGTCWNDLFNSSPCLCCHGPPRSTESG
PRF-06_6 MKFVTPLVLFCLCCVWMTVEGAAIREKELERGMEDILASLKTRQ-SCQLRDEVCNRKAGIRCCCGLTCHCTAAGLNRGC-TGTCWNDLFNSNPCL

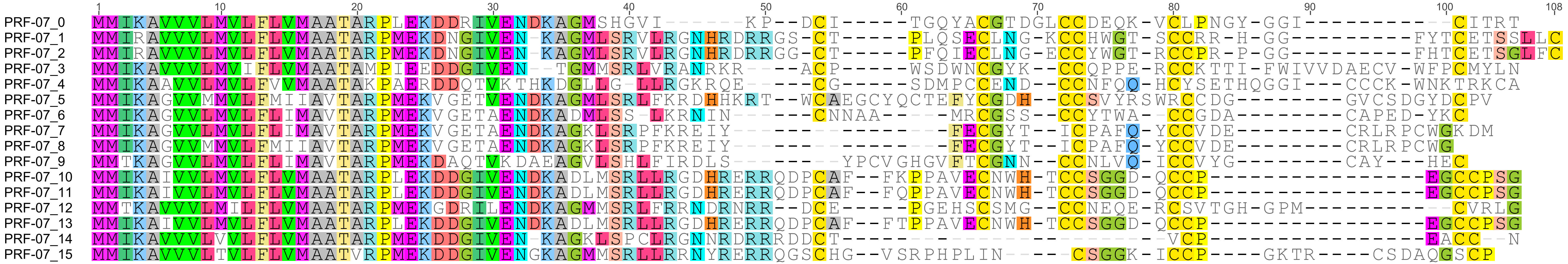
1
10
20
30
40
50
60
70
80
90
100
108
PRF-07_0
PRF-07_1
PRF-07_2
PRF-07_3
PRF-07_4
PRF-07_5
PRF-07_6
PRF-07_7
PRF-07_8
PRF-07_9
PRF-07_10
PRF-07_11
PRF-07_12
PRF-07_13
PRF-07_14
PRF-07_15

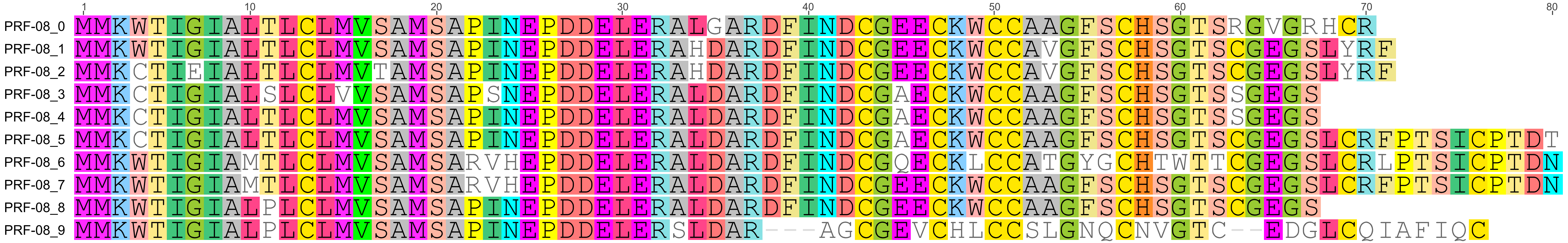
1 10 20 30 40 50 60 70 80
PRF-08_0 MMKWTIGIALTLCLMVSAMSAPINEPDDELERALGARDFINDCGEECKWCCAAGFSCHSGTSRGVGRHCR
PRF-08_1 MMKWTIGIALTLCLMVSAMSAPINEPDDELERAHDARDFINDCGEECKWCCAVGFSCHSGTSCGEGSLYRF
PRF-08_2 MMKCTIEIALTLCLMVTAMSAPINEPDDELERAHDARDFINDCGEECKWCCAVGFSCHSGTSCGEGSLYRF
PRF-08_3 MMKCTIGIALSLCLVVSAMSAPSNEPDDELERALDARDFINDCGAECKWCCAAGFSCHSGTSSGEGS
PRF-08_4 MMKCTIGIALTLCLMVSAMSAPINEPDDELERALDARDFINDCGAECKWCCAAGFSCHSGTSSGEGS
PRF-08_5 MMKCTIGIALTLCLMVSAMSAPINEPDDELERALDARDFINDCGAECKWCCAAGFSCHSGTSCGEGSLCRFPTSICPTDT
PRF-08_6 MMKWTIGIAMTLCLMVSAMSARVHEPDDELERALDARDFINDCGQECKLCCATGYGCHTWTTCGEGSLCRLPTSICPTDN
PRF-08_7 MMKWTIGIAMTLCLMVSAMSARVHEPDDELERALDARDFINDCGEECKWCCAAGFSCHSGTSCGEGSLCRFPTSICPTDN
PRF-08_8 MMKWTIGIALPLCLMVSAMSAPINEPDDELERALDARDFINDCGEECKWCCAAGFSCHSGTSCGEGS
PRF-08_9 MMKWTIGIALPLCLMVSAMSAPINEPDDELERSLDAR---AGCGEVCHLCCSLGNQCNVGTC--EDGLCQIAFIQC

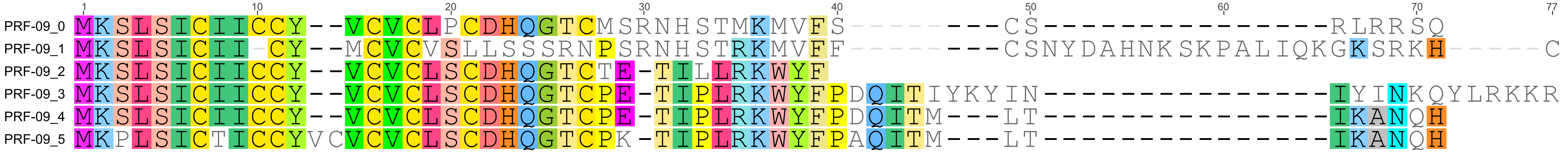
1 10 20 30 40 50 60 70 77
PRF-09_0 MKSLSICIICCY--VCVCLPCDHQGTCMSRNHSTMKMVFS-------CS----------------RLRRSQ
PRF-09_1 MKSLSICII-CY--MCVCVSLLSSSRNPSRNHSTRKMVFF-------CSNYDAHNKSKPALIQKGKSRKH-----C
PRF-09_2 MKSLSICIICCY--VCVCLSCDHQGTCTE-TILLRKWYF
PRF-09_3 MKSLSICIICCY--VCVCLSCDHQGTCPE-TIPLRKWYFPDQITIYKYIN----------------IYINKQYLRKKR
PRF-09_4 MKSLSICIICCY--VCVCLSCDHQGTCPE-TIPLRKWYFPDQITM---LT----------------IKANQH
PRF-09_5 MKPLSICTICCYVCVCVCLSCDHQGTCPK-TIPLRKWYFPAQITM---LT----------------IKANQH

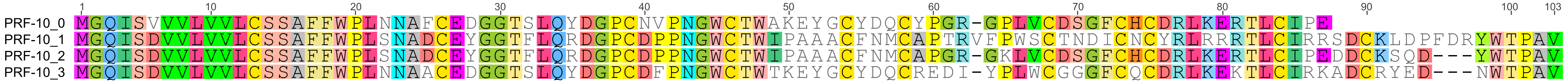
1 10 20 30 40 50 60 70 80 90 100 103
PRF-10_0 MGQISVVVLVVLCSSAFFWPLNNAFCEDGGTSLQYDGPCNVPNGWCTWAKEYGCYDQCYPGR-GPLVCDSGFCHCDRLKERTLCIPE
PRF-10_1 MGQISDVVLVVLCSSAFFWPLSNADCEYGGTFLQRDGPCDPPNGWCTWIPAAACFNMCAPTRVFPWSCTNDICNCYRLRRRTLCIRRSDCKLDPFDRYWTPAV
PRF-10_2 MGQISDVVLVVLCSSAFFWPLSNADCEYGGTFLQRDGPCDPPNGWCTWIPAAACFNMCAPGR-GKLVCDSGFCHCDRLKERTLCIPEDDCKSQD---YWTPAV
PRF-10_3 MGQISDVVLVVLCSSAFFWPLNNAACEDGGTSLQYDGPCDFPNGWCTWTKEYGCYDQCREDI-YPLWCGGGFCQCDRLKEKTLCIRKADCRYED---NWTPAV

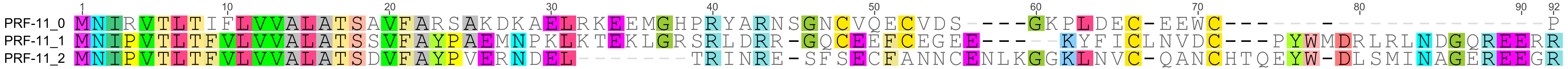
1 10 20 30 40 50 60 70 80 90 92
PRF-11_0 MNIRVTLTIFLVVALATSAVFARSAKDKAELRKEEMGHPRYARNSGNCVQECVDS----GKPLDEC-EEWC--------------------P
PRF-11_1 MNIPVTLTFVLVVALATSSVFAYPAEMNPKLKTEKLGRSRLDRR-GQCEEFCEGEE-----KYFICLNVDC---PYWMDRLRLNDGQREERR
PRF-11_2 MNIPVTLTFVLVVALATSDVFAYPVERNDEL-------TRINRE-SFSECFANNCENLKGGKLNVC-QANCHTQEYW-DLSMINAGEREEGR

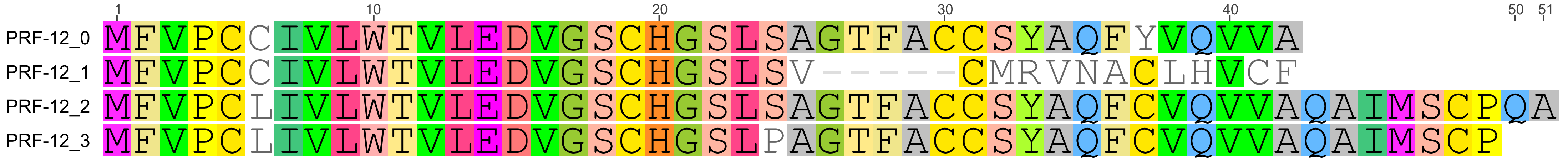
1
10
20
30
40
50
51
PRF-12_0 MFVPCCIVLWTVLEDVGSCHGSLSAGTFACCSYAQFYVQVVA
PRF-12_1 MFVPCCIVLWTVLEDVGSCHGSLSV-----CMRVNACLHVCF
PRF-12_2 MFVPCLIVLWTVLEDVGSCHGSLSAGTFACCSYAQFCVQVVAQAIMSCPQA
PRF-12_3 MFVPCLIVLWTVLEDVGSCHGSLPAGTFACCSYAQFCVQVVAQAIMSCP

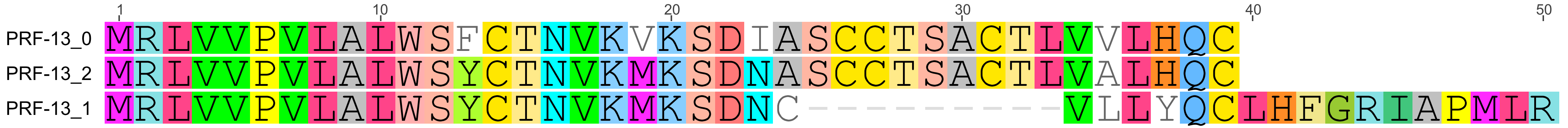
1
10
20
30
40
50
PRF-13_0 MRLVVPVLALWSFCTNVKVKSDIASCCTSACTLVVLHQC
PRF-13_2 MRLVVPVLALWSYCTNVKMKSDNASCCTSACTLVALHQC
PRF-13_1 MRLVVPVLALWSYCTNVKMKSDNC---------VLLYQCLHFGRIAPMLR

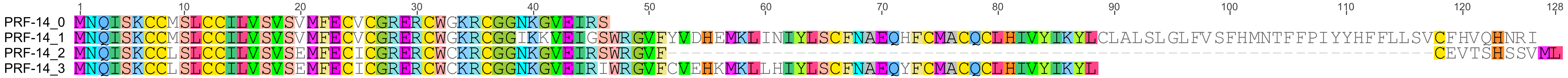
1 10 20 30 40 50 60 70 80 90 100 110 120 128
PRF-14_0 MNQISKCCMSLCCILVSVSVMFECVCGRERCWGKRCGGNKGVEIRS
PRF-14_1 MNQISKCCMSLCCILVSVSVMFECVCGRERCWGKRCGGIKKVEIGSWRGVFYVDHEMKLINIYLSCFNAEQHFCMACQCLHIVYIKYLCLALSLGLFVSFHMNTFFPIYYHFFLLSVCFHVQHNRI
PRF-14_2 MNQISKCCLSLCCILVSVSEMFECICGRERCWCKRCGGNKGVEIRSWRGVF--CEVTSHSSVML
PRF-14_3 MNQISKCCLSLCCILVSVSEMFECICGRERCWCKRCGGNKGVEIRIWRGVFCVEHKMKLLHIYLSCFNAEQYFCMACQCLHIVYIKYL

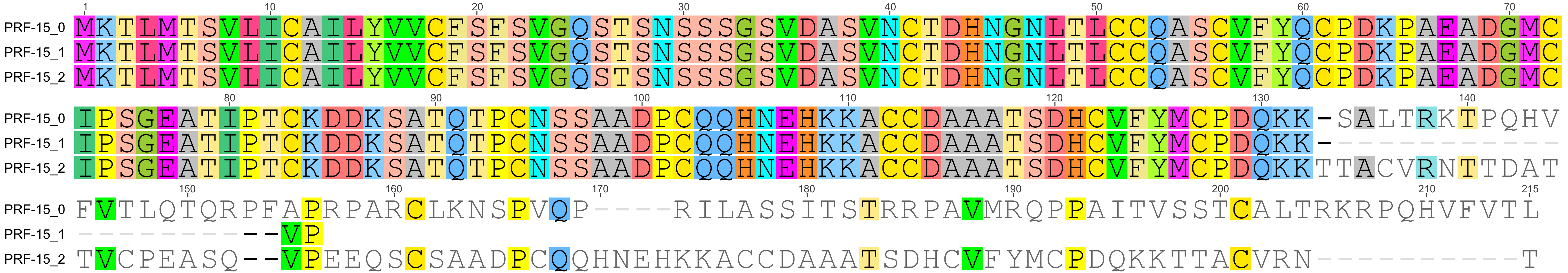
1 10 20 30 40 50 60 70
PRF-15_0 MKTLMTSVLICAILYVVCFSFSVGQSTSNSSSGSVDASVNCTDHNGNLTLCCQASCVFYQCPDKPAEADGMC
PRF-15_1 MKTLMTSVLICAILYVVCFSFSVGQSTSNSSSGSVDASVNCTDHNGNLTLCCQASCVFYQCPDKPAEADGMC
PRF-15_2 MKTLMTSVLICAILYVVCFSFSVGQSTSNSSSGSVDASVNCTDHNGNLTLCCQASCVFYQCPDKPAEADGMC
80 90 100 110 120 130 140
PRF-15_0 IPSGEATIPTCKDDKSATQTPCNSSAADPCQQHNEHKKACCDAAATSDHCVFYMCPDQKK-SALTRKTPQHV
PRF-15_1 IPSGEATIPTCKDDKSATQTPCNSSAADPCQQHNEHKKACCDAAATSDHCVFYMCPDQKK-
PRF-15_2 IPSGEATIPTCKDDKSATQTPCNSSAADPCQQHNEHKKACCDAAATSDHCVFYMCPDQKKTTACVRNTTDAT
150 160 170 180 190 200 210 215
PRF-15_0 FVTLQTQRPFAPRPARCLKNSPVQP----RILASSITSTRRPAVMRQPPAITVSSTCALTRKRPQHVFVTL
PRF-15_1 ----------VP
PRF-15_2 TVCPEASQ--VPEEQSCSAADPCQQHNEHKKACCDAAATSDHCVFYMCPDQKKTTACVRN---------T

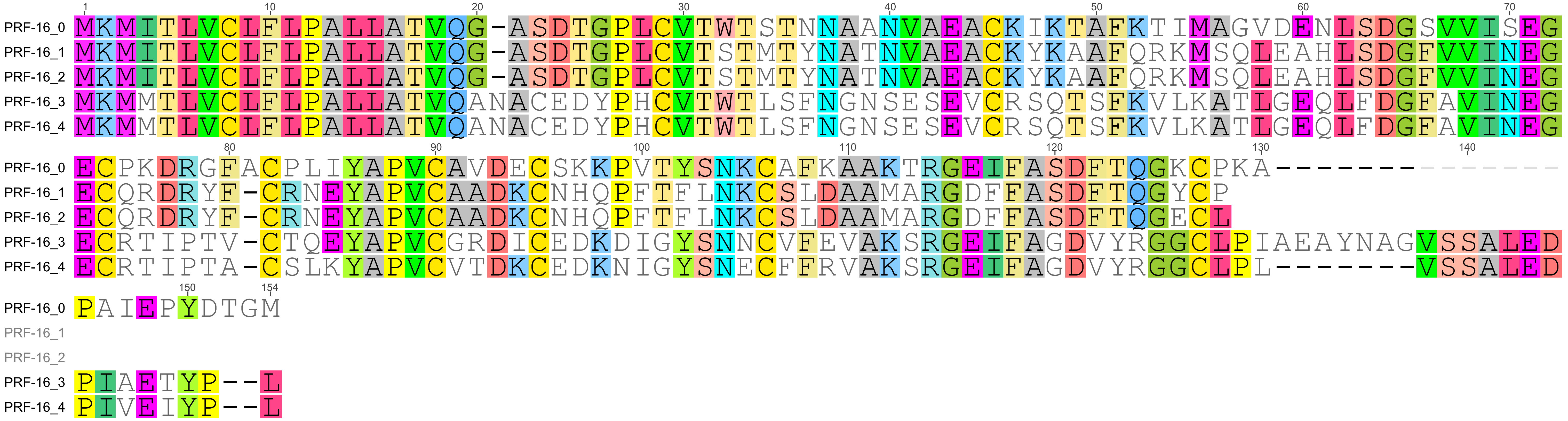
1 10 20 30 40 50 60 70
PRF-16_0 MKMITLVCLFLPALLATVQG-ASDTGPLCVTWTSTNNAANVAEACKIKTAFKTIMAGVDENLSDGSVVISEG
PRF-16_1 MKMITLVCLFLPALLATVQG-ASDTGPLCVTSTMTYNATNVAEACKYKAAFQRKMSQLEAHLSDGFVVINEG
PRF-16_2 MKMITLVCLFLPALLATVQG-ASDTGPLCVTSTMTYNATNVAEACKYKAAFQRKMSQLEAHLSDGFVVINEG
PRF-16_3 MKMMTLVCLFLPALLATVQANACEDYPHCVTWTLSFNGNSESEVCRSQTSFKVLKATLGEQLFDGFAVINEG
PRF-16_4 MKMMTLVCLFLPALLATVQANACEDYPHCVTWTLSFNGNSESEVCRSQTSFKVLKATLGEQLFDGFAVINEG
80 90 100 110 120 130 140
PRF-16_0 ECPKDRGFACPLIYAPVCAVDECSKKPVTYSNKCAFKAAKTRGEIFASDFTQGKCPKA--------
PRF-16_1 ECQRDRYF-CRNEYAPVCAADKCNHQPFTFLNKCSLDAAMARGDFFASDFTQGYCP
PRF-16_2 ECQRDRYF-CRNEYAPVCAADKCNHQPFTFLNKCSLDAAMARGDFFASDFTQGECL
PRF-16_3 ECRTIPTV-CTQEYAPVCGRDICEDKDIGYSNNCVFEVAKSRGEIFAGDVYRGGCLPIAEAYNAGVSSALED
PRF-16_4 ECRTIPTA-CSLKYAPVCVTDKCEDKNIGYSNECFFRVAKSRGEIFAGDVYRGGCLPL--------VSSALED
150 154
PRF-16_0 PAIEPYDTGM
PRF-16_1
PRF-16_2
PRF-16_3 PIAETYP--L
PRF-16_4 PIVEIYP--L

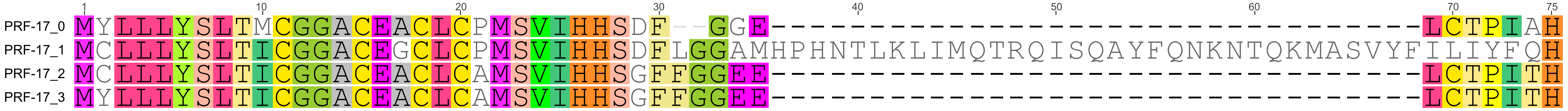
1 10 20 30 40 50 60 70 75
PRF-17_0 MYLLLYSLTMCGGACEACLCPMSVIHHSDF--GGE---------------------------------LCTPIAH
PRF-17_1 MCLLLYSLTICGGACEGCLCPMSVIHHSDFLGGAMHPHNTLKLIMQTRQISQAYFQNKNTQKMASVYFILIYFQH
PRF-17_2 MCLLLYSLTICGGACEACLCAMSVIHHSGFFGGEE---------------------------------LCTPITH
PRF-17_3 MYLLLYSLTICGGACEACLCAMSVIHHSGFFGGEE---------------------------------LCTPITH

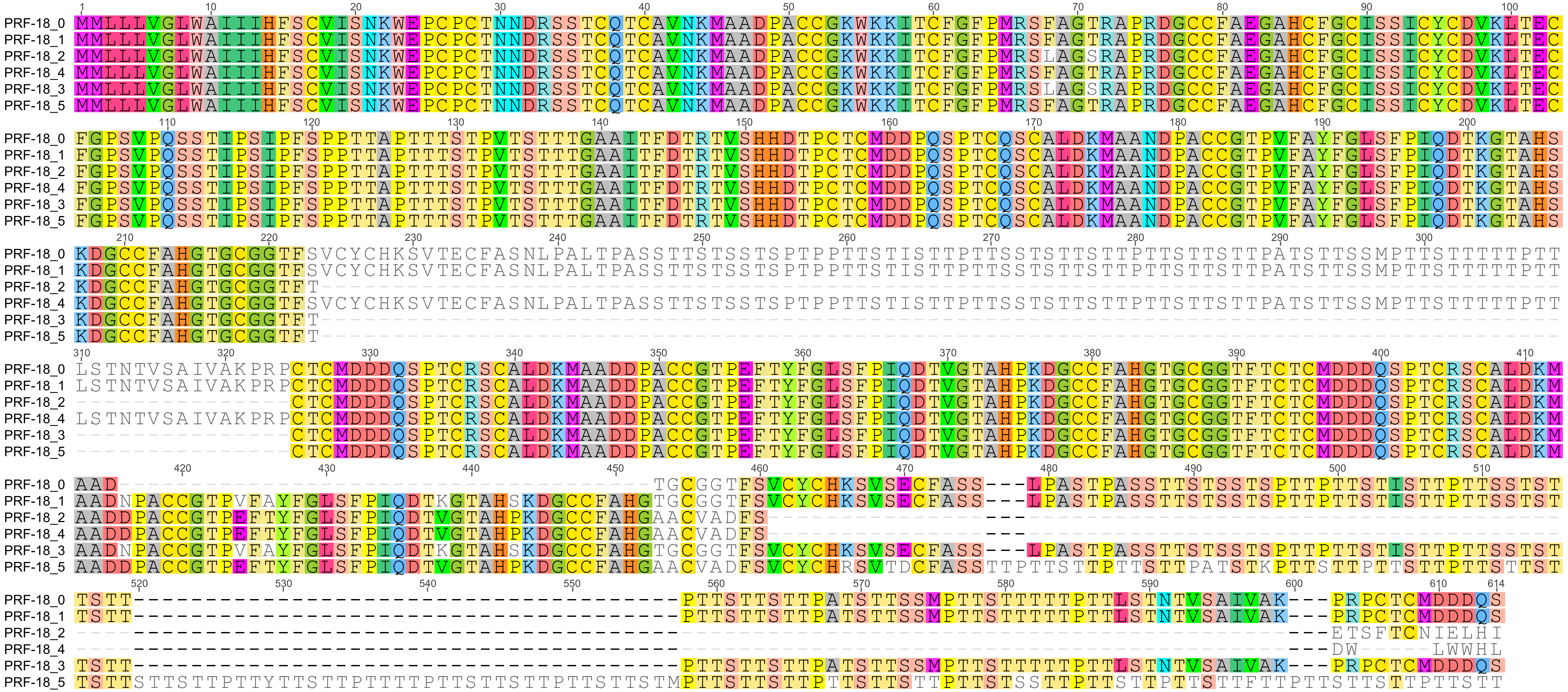
1 10 20 30 40 50 60 70 80 90 100
PRF-18_0 MMLLLVGLWAIIIHFSCVISNKWEPCPCTNNDRSSTCQTCAVNKMAADPACCGKWKKITCFGFPMRSFAGTRAPRDGCCFAEGAHCFGCISSICYCDVKLTEC
PRF-18_1 MMLLLVGLWAIIIHFSCVISNKWEPCPCTNNDRSSTCQTCAVNKMAADPACCGKWKKITCFGFPMRSFAGTRAPRDGCCFAEGAHCFGCISSICYCDVKLTEC
PRF-18_2 MMLLLVGLWAIIIHFSCVISNKWEPCPCTNNDRSSTCQTCAVNKMAADPACCGKWKKITCFGFPMRSLAGSRAPRDGCCFAEGAHCFGCISSICYCDVKLTEC
PRF-18_4 MMLLLVGLWAIIIHFSCVISNKWEPCPCTNNDRSSTCQTCAVNKMAADPACCGKWKKITCFGFPMRSFAGTRAPRDGCCFAEGAHCFGCISSICYCDVKLTEC
PRF-18_3 MMLLLVGLWAIIIHFSCVISNKWEPCPCTNNDRSSTCQTCAVNKMAADPACCGKWKKITCFGFPMRSLAGSRAPRDGCCFAEGAHCFGCISSICYCDVKLTEC
PRF-18_5 MMLLLVGLWAIIIHFSCVISNKWEPCPCTNNDRSSTCQTCAVNKMAADPACCGKWKKITCFGFPMRSFAGTRAPRDGCCFAEGAHCFGCISSICYCDVKLTEC
110 120 130 140 150 160 170 180 190 200
PRF-18_0 FGPSVPQSSTIPSIPFSPPTTAPTTTSTPVTSTTTGAAITFDTRTVSHHDTPCTCMDDPQSPTCQSCALDKMAANDPACCGTPVFAYFGLSFPIQDTKGTAHS
PRF-18_1 FGPSVPQSSTIPSIPFSPPTTAPTTTSTPVTSTTTGAAITFDTRTVSHHDTPCTCMDDPQSPTCQSCALDKMAANDPACCGTPVFAYFGLSFPIQDTKGTAHS
PRF-18_2 FGPSVPQSSTIPSIPFSPPTTAPTTTSTPVTSTTTGAAITFDTRTVSHHDTPCTCMDDPQSPTCQSCALDKMAANDPACCGTPVFAYFGLSFPIQDTKGTAHS
PRF-18_4 FGPSVPQSSTIPSIPFSPPTTAPTTTSTPVTSTTTGAAITFDTRTVSHHDTPCTCMDDPQSPTCQSCALDKMAANDPACCGTPVFAYFGLSFPIQDTKGTAHS
PRF-18_3 FGPSVPQSSTIPSIPFSPPTTAPTTTSTPVTSTTTGAAITFDTRTVSHHDTPCTCMDDPQSPTCQSCALDKMAANDPACCGTPVFAYFGLSFPIQDTKGTAHS
PRF-18_5 FGPSVPQSSTIPSIPFSPPTTAPTTTSTPVTSTTTGAAITFDTRTVSHHDTPCTCMDDPQSPTCQSCALDKMAANDPACCGTPVFAYFGLSFPIQDTKGTAHS
210 220 230 240 250 260 270 280 290 300
PRF-18_0 KDGCCFAHGTGCGGTFSVCYCHKSVTECFASNLPALTPASSTTSTSSTSPTPPTTSTISTTPTTSSTSTTSTTPTTSTTSTTPATSTTSSMPTTSTTTTTPTT
PRF-18_1 KDGCCFAHGTGCGGTFSVCYCHKSVTECFASNLPALTPASSTTSTSSTSPTPPTTSTISTTPTTSSTSTTSTTPTTSTTSTTPATSTTSSMPTTSTTTTTPTT
PRF-18_2 KDGCCFAHGTGCGGTFT
PRF-18_4 KDGCCFAHGTGCGGTFSVCYCHKSVTECFASNLPALTPASSTTSTSSTSPTPPTTSTISTTPTTSSTSTTSTTPTTSTTSTTPATSTTSSMPTTSTTTTTPTT
PRF-18_3 KDGCCFAHGTGCGGTFT
PRF-18_5 KDGCCFAHGTGCGGTFT
310 320 330 340 350 360 370 380 390 400 410
PRF-18_0 LSTNTVSAIVAKPRPCTCMDDDQSPTCRSCALDKMAADDPACCGTPEFTYFGLSFPIQDTVGTAHPKDGCCFAHGTGCGGTFTCTCMDDDQSPTCRSCALDKM
PRF-18_1 LSTNTVSAIVAKPRPCTCMDDDQSPTCRSCALDKMAADDPACCGTPEFTYFGLSFPIQDTVGTAHPKDGCCFAHGTGCGGTFTCTCMDDDQSPTCRSCALDKM
PRF-18_2 CTCMDDDQSPTCRSCALDKMAADDPACCGTPEFTYFGLSFPIQDTVGTAHPKDGCCFAHGTGCGGTFTCTCMDDDQSPTCRSCALDKM
PRF-18_4 LSTNTVSAIVAKPRPCTCMDDDQSPTCRSCALDKMAADDPACCGTPEFTYFGLSFPIQDTVGTAHPKDGCCFAHGTGCGGTFTCTCMDDDQSPTCRSCALDKM
PRF-18_3 CTCMDDDQSPTCRSCALDKMAADDPACCGTPEFTYFGLSFPIQDTVGTAHPKDGCCFAHGTGCGGTFTCTCMDDDQSPTCRSCALDKM
PRF-18_5 CTCMDDDQSPTCRSCALDKMAADDPACCGTPEFTYFGLSFPIQDTVGTAHPKDGCCFAHGTGCGGTFTCTCMDDDQSPTCRSCALDKM
420 430 440 450 460 470 480 490 500 510
PRF-18_0 AAD TGCGGTFSVCYCHKSVSECFASS---LPASTPASSTTSTSSTSPTTPTTSTISTTPTTSSTST
PRF-18_1 AADNPACCGTPVFAYFGLSFPIQDTKGTAHSKDGCCFAHGTGCGGTFSVCYCHKSVSECFASS---LPASTPASSTTSTSSTSPTTPTTSTISTTPTTSSTST
PRF-18_2 AADDPACCGTPEFTYFGLSFPIQDTVGTAHPKDGCCFAHGAACVADFS ---
PRF-18_4 AADDPACCGTPEFTYFGLSFPIQDTVGTAHPKDGCCFAHGAACVADFS ---
PRF-18_3 AADNPACCGTPVFAYFGLSFPIQDTKGTAHSKDGCCFAHGTGCGGTFSVCYCHKSVSECFASS---LPASTPASSTTSTSSTSPTTPTTSTISTTPTTSSTST
PRF-18_5 AADDPACCGTPEFTYFGLSFPIQDTVGTAHPKDGCCFAHGAACVADFSVCYCHRSVTDCFASSTTPTTSTTPTTSTTPATSTKPTTSTTPTTSTTPTTSTTST
520 530 540 550 560 570 580 590 600 610 614
PRF-18_0 TSTT------------------------------------PTTSTTSTTPATSTTSSMPTTSTTTTTPTTLSTNTVSAIVAK---PRPCTCMDDDQS
PRF-18_1 TSTT------------------------------------PTTSTTSTTPATSTTSSMPTTSTTTTTPTTLSTNTVSAIVAK---PRPCTCMDDDQS
PRF-18_2 ETSFTCNIELHI
PRF-18_4 ------------------------------------ ---DW LWWHL
PRF-18_3 TSTT------------------------------------PTTSTTSTTPATSTTSSMPTTSTTTTTPTTLSTNTVSAIVAK---PRPCTCMDDDQS
PRF-18_5 TSTTSTTSTTPTTYTTSTTPTTTTPTTSTTSTTPTTSTTSTMPTTSTTSTTPTSTTSTTPTTSTSSTTPTTSTTPTTSTTFTTPTTSTTSTTPTTSTT

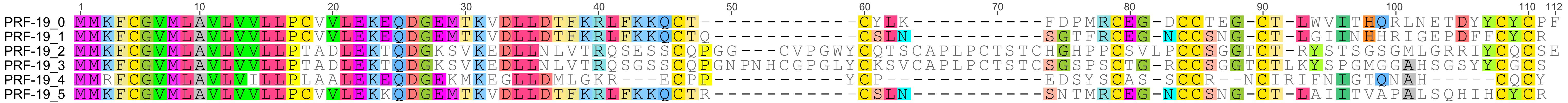
1 10 20 30 40 50 60 70 80 90 100 110 112
PRF-19_0 MMKFCGVMLAVLVVLLPCVVLEKEQDGEMTKVDLLDTFKRLFKKQCT CYLK FDPMRCEG DCCTEG CT LWVITHQRLNETDYCYCPF
PRF-19_1 MMKFCGVMLAVLVVLLPCVVLEKEQDGEMTKVDLLDTFKRLFKKQCTQ CSLN SGTFRCEG NCCSNG CT LGIINHHRIGEPDFFCYCR
PRF-19_2 MMKFCGVMLAVLVVLLPTADLEKTQDGKSVKEDLLNLVTRQSESSCQPGG CVPGWYCQTSCAPLPCTSTCHGHPPCSVLPCCSGGTCT RYSTSGSGMLGRRIYCQCSE
PRF-19_3 MMKFCGVMLAVLVVLLPTADLEKTQDGKSVKEDLLNLVTRQSGSSCQPGNPNHCGPGLYCKSVCAPLPCTSTCSGSPSCTG RCCSGGTCTLKYSPGMGGAHSGSYYCGCS
PRF-19_4 MMRFCGVMLAVLVILLPLAALEKEQDGEKMKEGLLDMLGKR ECPP YCP EDSYSCAS SCCR NCIRIFNIGTQNAH CQCY
PRF-19_5 MMKFCGVMLAVLVVLLPCVVLEKKQDGEMTKVDLLDTFKRLFKKQCTR CSLN SNTMRCEG NCCSNG CT LAIITVAPALSQHIHCYCR

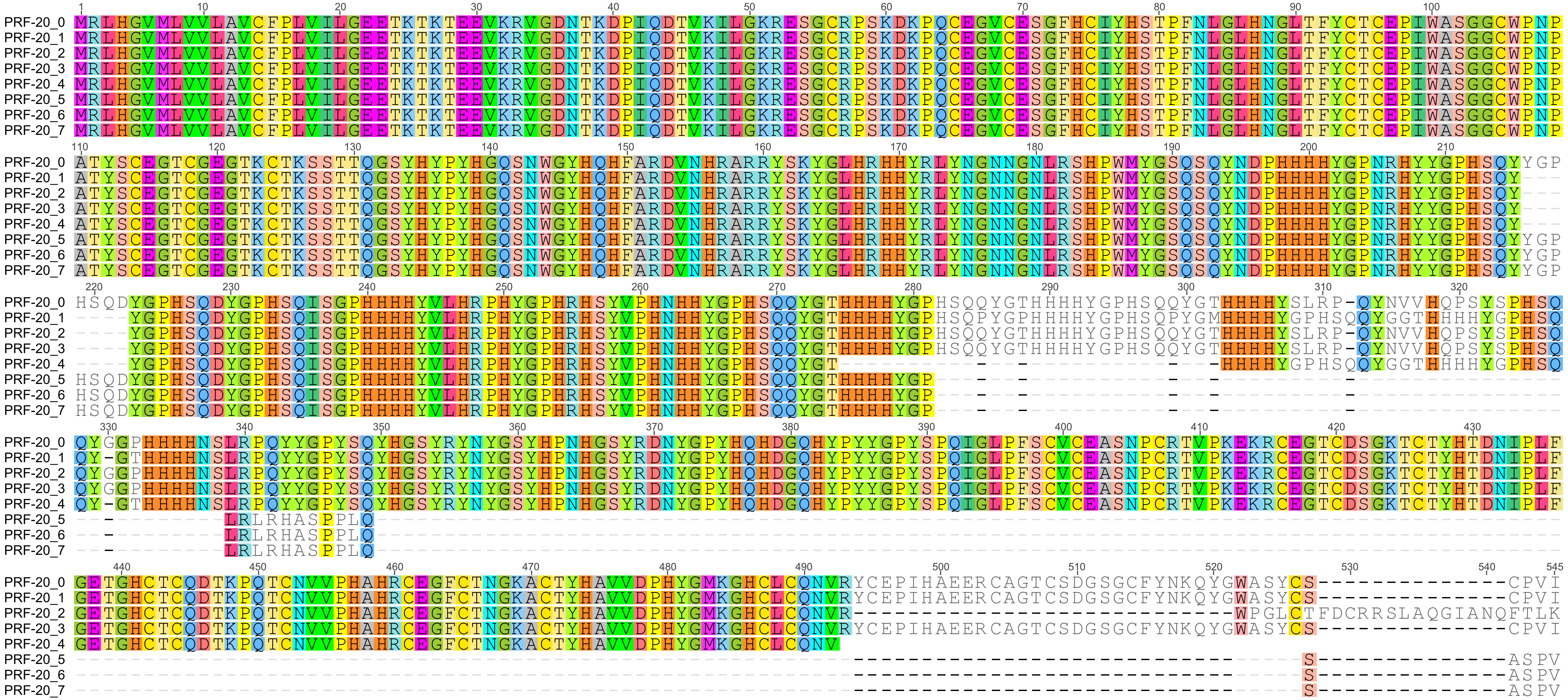
PRF-20_0
PRF-20_1
PRF-20_2
PRF-20_3
PRF-20_4
PRF-20_5
PRF-20_6
PRF-20_7
